# Aromatic Polymethacrylates from Lignin‐Based Feedstock: Synthesis, Thermal Properties, Life‐Cycle Assessment and Toxicity

**DOI:** 10.1002/cssc.202401239

**Published:** 2024-10-23

**Authors:** Rauno Sedrik, Olivier Bonjour, Nariê Rinke Dias de Souza, Alina Ismagilova, Iris Tamsalu, Veljo Kisand, Francesco Cherubini, Patric Jannasch, Lauri Vares

**Affiliations:** ^1^ Institute of Technology University of Tartu Nooruse 1 Tartu 50411 Estonia; ^2^ Department of Chemistry Lund University Box 124 221 00 Lund Sweden; ^3^ Industrial Ecology Programme Department of Energy and Process Engineering Norwegian University of Science and Technology Trondheim Norway

**Keywords:** Life cycle assessment, Lignin, Polymers, Renewable resources, Toxicology

## Abstract

There is currently a great need for rigid, high‐performance and processable bio‐based polymers and plastics as alternatives to the fossil‐based materials used today. Here, we report on the straightforward synthesis and polymerization of lignin‐derived methacrylate monomers based on the methyl esters of 4‐hydroxybenzoic, vanillic, and syringic acid, respectively. The corresponding homopolymethacrylates exhibit high glass transition temperatures (*T*_g_s) at 106, 128, and 197 °C, respectively. Rheological properties and thermal stability up to at least 277 °C indicate that these polymers are melt‐processable. In addition, copolymers with methyl methacrylate are prepared to further vary and tune the polymer properties. An integrated ex‐ante and prospective life‐cycle assessment of key environmental impact parameters indicates similar or only slightly higher values compared to well‐established fossil‐based methyl methacrylate. Moreover, the toxicity towards human HeLa cell lines compares well with that of poly(methyl methacrylate). Hence, the potential availability of lignin‐derived acids, combined with the straightforward and potentially upscalable monomer synthesis, make these rigid polymers appealing alternatives towards bio‐based high‐*T*_g_ thermoplastic materials with low toxicity.

## Graphical abstract

A scalable method was developed to convert lignin‐based aromatic acids into methacrylate monomers. Subsequently, polymethacrylates with glass transition temperatures close to 200 °C were prepared by radical polymerization. Life cycle and toxicity evaluation showed results competitive with fossil‐based methyl methacrylate.

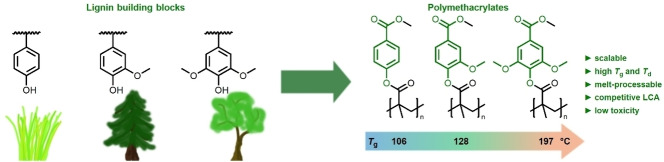

## Introduction

Lowering the environmental impact of the fossil raw material based polymer industry is critical in our path toward a more sustainable economy.^[1]^ To phase out the fossil feedstock, biomass comes into play as an alternative source for raw materials. Several new bio‐based polymers have been prepared, and many can compete with conventional polymers based on properties.^[2]^ However, their overall environmental impact is still typically higher compared to commercial fossil‐based materials, especially in categories other than global warming potential.^[3]^ For example, bio‐polyethylene (PE) has been reported to have a lower carbon footprint compared to fossil HDPE if produced using renewable energy and high transport emissions are avoided (−1.0 and 2.6 kg CO_2_eq/kg for bio‐PE and fossil‐PE, respectively).^[4]^ Still, land use change can make bio‐PE less favorable compared to fossil PE, as replacing forests with sugar cane plantations results in a higher carbon footprint (3.6 kg CO_2_eq/kg), and additionally, bio‐PE has about ten times higher impacts for eutrophication and acidification.^[5]^ Hence, developing bio‐based polymers with competitive environmental impact is a reoccurring challenge and the proper life‐cycle assessment (LCA) should preferably comprise several impact categories.^[6]^

**Scheme 1:**
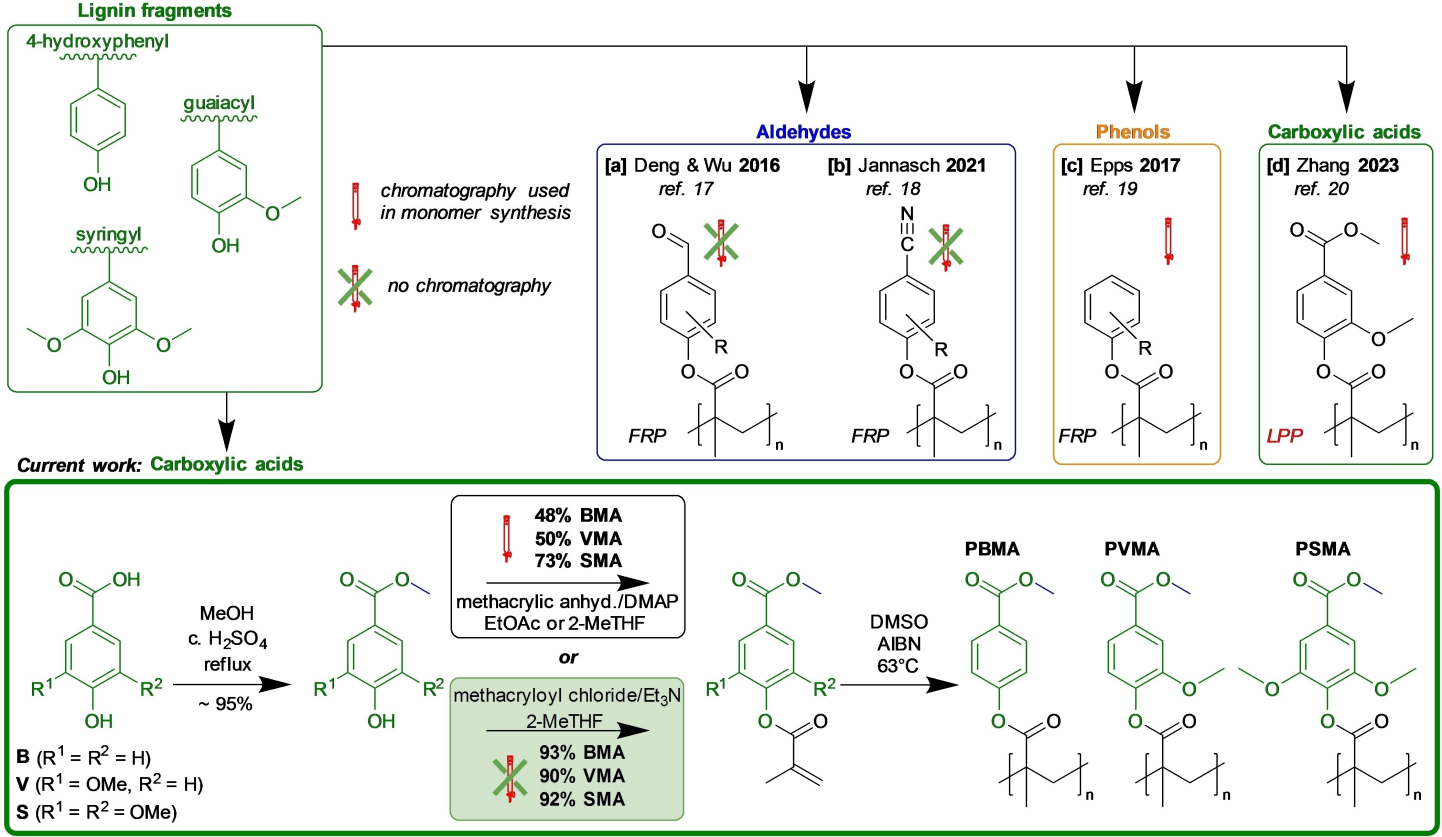
Previously reported lignin‐based polymethacrylates and those presented in the current work.

The planet′s most abundant and cheapest organic carbon source is lignocellulosic biomass.^[7]^ While cellulose and hemicellulose have found wider use, the third main lignocellulosic component, lignin, has been mainly used as a low‐efficiency fuel.^[8]^ Lignin is a biopolymer incorporating three main types of units,i. e., syringyl, guaiacyl, and 4‐hydroxyphenyl, which have two, one, and none methoxy groups attached to the phenyl ring, respectively (Scheme 1). The ratio between these three units depends on the type of lignin source, e. g., hardwoods contain more syringyl units than softwoods. Lignin compositions also differ between plant species and even parts of the same plant, leading to a large variations in the lignin structures.[^9^, ^10^]

Obtaining homogeneous and well‐defined plastics from lignin is difficult due to its intrinsic recalcitrance. Therefore, it is necessary to process lignin further to obtain suitable building blocks for polymer chemistry.[^11^, ^12^, ^13^] Currently, the only large‐scale chemical produced from lignin is vanillin.^[14]^ Hence, in the field of thermoplastic poly(meth)acrylates, mostly vanillin, but also other potentially lignin derived aromatic aldehydes, such as 4‐hydroxymethyl‐ and syringic aldehyde, have been investigated as a polymer building blocks.[^11^, ^15^, ^16^, ^17^, ^18^, ^19^, ^20^]

For example, Deng and Wu et al. prepared monomers by attaching methacrylate and acrylate functionalities to the phenolic hydroxyl groups in vanillin and syringyl aldehyde (Scheme 1a).^[17]^ The corresponding polymethacrylates exhibited *T*_g_s up to 180 °C. We have recently used a similar strategy but, in addition, converted the aldehyde functional groups in vanillin, syringaldehyde, and 4‐hydroxybenzaldehyde into nitrile groups, which after methacrylation and free‐radical polymerization afforded polymethacrylates with *T*_g_s, up to 238 °C (Scheme 1b).^[18]^

Other potentially lignin‐derived aromatic phenols have also been investigated. Epps et al. have studied various monomers based on guaiacol, 4‐ethylguaiacol, and creosol, where methacrylate groups were attached to the phenolic hydroxyl groups. Polymethacrylates obtained by free‐radical polymerization of these monomers showed *T*_g_s from 116 to 139 °C.^[15]^ The same group has also reported on a series of polymethacrylates containing various dimethoxyphenyl substituents (Scheme 1c)^[19]^ and syringyl methacrylate homo‐ and copolymers with *T*_g_s up to 205 °C.^[16]^

Typically, thermoplastic polymethacrylates from lignin‐derived building blocks are amorphous and exhibit *T*_g_s that significantly exceed that of, e. g., polystyrene (*T*_g_=100 °C) and PMMA (*T*_g_=105 °C) offering a competitive bio‐based alternative. The high *T*_g_s of these aromatic polymethacrylates are generally rationalized by a high chain rotational barrier and strong *π*‐*π* stacking interactions of the aromatic units.^[21]^

Using a slightly different approach, we have recently attached vanillic‐ and other lignin‐related benzoic acids to widely available isosorbide.^[22]^ Methacrylation of these compounds, followed by free‐radical polymerization (FRP), afforded polymethacrylates with *T*_g_s from 80 to 168 °C. This was up to 60 °C higher than the corresponding isosorbide polymethacrylates with fully aliphatic side chains.[^23^, ^24^]

Interestingly, methacrylates derived directly from vanillic‐, syringic‐, and 4‐hydroxyphenolic acids have not been properly evaluated for preparing bio‐based polymethacrylates, except one example by Zhang, where the vanillic acid derived methacrylate was polymerized via Lewis pair polymerization (LPP) using very moisture sensitive and rather hard‐to‐access Al(C_6_F_5_)_3_/P(NI^*i*^Pr)Ph_2_ Lewis pair (Scheme 1d).^[20]^ Such aromatic carboxylic acids, however, are easily accessible via oxidation of corresponding aldehydes.^[25]^ Moreover, studies to obtain such acids directly from lignin have recently emerged, further facilitating their potential availability.[^26^, ^27^]

In the present work, we report on a potentially upscalable and chromatography‐free process to obtain methacrylate monomers from vanillic, syringic, and 4‐hydroxybenzoic acid, respectively. Each methacrylate monomer was polymerized using a conventional free‐radical mechanism into corresponding homopolymers and copolymers with methyl methacrylate (MMA). To establish important structure‐property relationships, these polymethacrylates were characterized with respect to structure, molecular weight, thermal stability, thermal transitions, and rheological properties. Moreover, this initial study of these materials is complemented with a life‐cycle assessment (LCA) of three impact parameters (i. e., global warming potential, terrestrial acidification, and freshwater eutrophication) and the cytotoxicity evaluation towards human HeLa cells.

## Results and Discussion

### Monomer Synthesis

The synthesis of the methacrylate monomers included two standard steps, i. e., the conversion of the corresponding benzoic acid derivative into the methyl ester, and the methacrylation of the phenolic hydroxyl group (Scheme 1). Initially, we evaluated the use of acidic catalysis for both the esterification of the carboxylic group with MeOH and the methacrylation of the hydroxyl functionality using methacrylic acid. However, the latter reaction resulted in low yields, probably due to the instability of the methacrylic derivatives at elevated temperatures. Hence, we decided to use acidic catalysis for the esterification of benzoic acids in the first step but needed to find alternatives for the subsequent methacrylation step.

Trans‐esterification using MMA was assessed, but the yields obtained were low. Next, combining previously described methods, we tried methacrylic anhydride as the methacrylate source.[^28^, ^29^] However, this approach afforded incomplete conversion and significant amounts of byproducts (~30 %), resulting in low to moderate yields, i. e., 48, 50, and 73 % for **BMA**, **VMA**, and **SMA**, respectively. Moreover, chromatographic separation was generally needed to obtain the pure monomers. Based on ^1^H NMR analysis, these side products contained dimers and oligomers from condensation reactions between the benzoic acids and the free hydroxyl groups. Additionally, there might be some side reactions involving the aromatic ring itself. These side products were less prevalent with the **SMA** monomer, probably because the aromatic ring was sterically more protected towards side reactions. This allowed **SMA** to be obtained in a 65 % yield by crystallization in ethyl acetate after washing the crude mixture with brine and filtration through a thin layer of silica.

To further improve the methacrylation step, we evaluated using methacryloyl chloride^[20]^ instead of methacrylic anhydride. Acylations with acid chlorides are typically carried out in chlorinated solvents such as CH_2_Cl_2_. However, in the present case, we replaced this solvent with bio‐based 2‐MeTHF without any noticeable negative impact on the reaction outcome. Hence, we obtained yields of 93, 90, and 92 % for **BMA**, **VMA**, and **SMA**, respectively, without chromatographic purification. This proved to be a significant increase in the yield of all the monomers. It should be noted that the high purity of methacryloyl chloride was essential to reach such high yields.

### Polymerization

Initially, we evaluated polymerizations in a range of solvents such as EtOAc, chloroform, and DMSO, and in biobased alternatives such as GVL and 2‐MeTHF,^[30]^ to identify the most suitable one for the polymerization of these monomers (Table S1). The consistently best polymerization results were reached using DMSO, which was selected as the polymerization medium. The only observed drawback of the polymers prepared in DMSO was the relatively high dispersity (*Đ*), between 3.6 and 4.1. Notably, high *Đ* values have been previously reported for polymethacrylates prepared in DMSO.^[31]^

Using DMSO as the polymerization medium enabled us to obtain all three homopolymers with *M*_n_ values in a narrow range (i. e., between 54–62 kg/mol, Table 1, entries 1, 3, 4). The isolated yields in the **PBMA, PVMA**, and **PSMA** polymerizations were 46, 84, and 77 %, respectively. **PBMA‐2** was prepared on a scale 5 times larger than PBMA and was obtained with an *M*_n_ of 121 kg/mol, twice that of **PBMA**. Most probably, this was an effect of the difference in scale. In addition, **PBMA‐2** was precipitated twice, which may have resulted in the removal of some low‐*M*_n_ fraction of the polymer, leading to a higher average *M*_n_ value and lower polydispersity compared to other homopolymers. The lower isolated yield of **PSMA** may be explained by the lower reactivity of the C=C double bond due to the additional electron‐donating effect or by the additional steric hindrance from the two methoxy groups in the aromatic ring.

**Table 1:** Polymerization and thermal data of homo‐ and copolymers prepared in DMSO.

Entry	Polymer	AIBN (mol %)	MMA content (mol%)		*M*_n_^[b]^ (kg/mol)	*Đ* ^[b]^	Isolated yield (%)	*T*_g_^[c]^ (°C)	*T*_d,95%_^[d]^ (°C)
			Target	Obtained^[a]^					
1	PBMA	0.500	–	–	62	3.6	71	106	277
2	PBMA‐2	0.500	–	–	121	3.1	77	133	280
3	PVMA	0.500	–	–	60	3.6	84	128	313
4	PSMA	0.500	–	–	54	3.6	46	197	307
5	PVMMA‐25	0.125	25	22	236	1.9	91	134	292
6	PVMMA‐50	0.250	50	44	93	2.6	78	127	281
7	PVMMA‐75	0.375	75	74	69	2.7	79	125	271
8	PSMMA‐75	0.375	75	69	54	2.8	83	139	258

^[a]^ Determined from ^1^H NMR spectra. ^[b]^ Measured by SEC in THF. ^[c]^ Measured using DSC. ^[d]^ Thermal degradation temperature at a 5 % mass loss.

Copolymerizations of the lignin‐derived methacrylates with MMA were also studied (Table 1, entries 5–8). Analysis of the ^1^H NMR spectra of these statistical copolymers showed that the measured MMA:**VMA** and MMA:**SMA** ratios only deviated slightly (1–6 %) from the target values used in polymerizations. The copolymers had *M_n_* values ranging from 54 to 236 kg/mol, corresponding to the AIBN concentration used, i. e., higher concentration lead to lower *M*_n_ values. These AIBN concentrations were chosen to maintain a constant AIBN to stabilizer ratio, as the stabilizer was not removed from MMA. Similar to the results obtained with the homopolymers, copolymer **PSMMA‐75** had a lower molecular weight and yield compared to **PVMMA‐75**. Contrary to our previous studies on isosorbide methacrylates,^[24]^ the lower AIBN concentrations did not decrease the isolated yields of the polymers, indicating high monomer conversions. We also noticed slightly lower *Đ* values for the copolymers compared to the homopolymers.

### Thermal Properties

**Figure 1:**
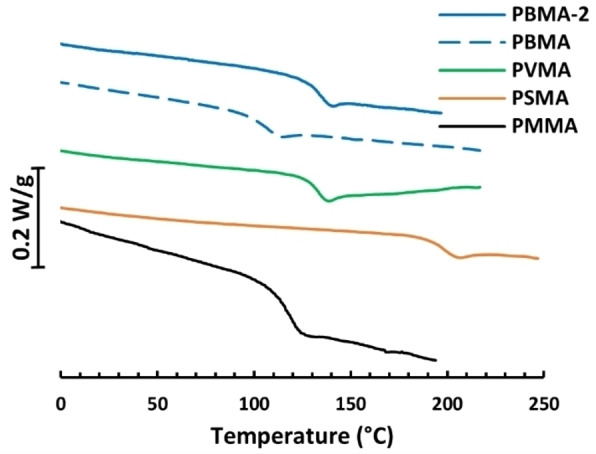
DSC thermograms of samples PBMA, PBMA‐2, PVMA, PSMA, and PMMA.

Thermogravimetric analysis (TGA) of the homopolymer samples showed a decomposition temperature (*T*_d,_95 %) ranging from 277 to 313 °C (Table 1 and Figure S15), indicating a high thermal stability of these materials. Differential scanning calorimetry of these samples showed, as expected, that they were fully amorphous and displayed single glass transitions (Figure 1). The *T*_g_ values of the homopolymethacrylates increased with the number of methoxy groups per benzene ring. At *M*_n_ approx. 60 kg/mol, the values were 106, 128 and 197 °C for the samples with none, one and two −OCH_3_ groups per ring, respectively. This finding was in accordance with our previous study on nitrile‐containing polymethacrylates.^[18]^

The *T*_g_ values of the two samples derived from 4‐hydroxybenzoic acid were 106 and 133 °C for **PBMA** (*M_n_* =62 kg/mol) and **PBMA‐2** (*M_n_* =121 kg/mol), respectively, which hinted that *T*_g_^∞^ in Flory–Fox equation (i.e., *T*_g_ at infinite *M*_n_) is close to 161 °C. The *T*_g_ value of **PVMA** was found at 128 °C, which agreed very well with the literature value previously reported by Zhang et al. (126 °C), although this value was obtained with *M_n_* =300 kg/mol.^[20]^ **PSMA** with two methoxy groups on the aromatic ring reached a very high *T*_g_ value (197 °C). This was higher than for the previously reported structurally similar syringaldehyde polymethacrylate (180 °C),^[17]^ but lower than 2,6‐dimethoxyphenyl polymethacrylate (210 °C),^[20]^ indicating that the *para*‐substituted methyl ester groups of the present materials have a lower increasing effect on *T*_g_ compared to aldehyde group in the same position. Still, **PSMA** reached an impressive *T*_g_ value for a bio‐based polymer, and would be a good candidate for high‐performance polymer applications, such as package materials hot‐fill applications. Moreover, the temperature window between *T*_g_ and *T*_d,95%_, was more than 100 °C, indicating potential melt processability.

The homopolymers showed *T*_g_s well above 100 °C and were rigid, fully amorphous thermoplastics. This indicated that the aromatic methacrylate monomers may be employed to improve the thermal properties of other polymers through copolymerization. Consequently, we carried out copolymerizations with MMA, which is widely used in the polymer industry. Again, DMSO was chosen as the polymerization medium in these experiments because of the consistent results obtained in the homopolymerizations.

TGA showed that the copolymers also exhibited relatively high thermal stability. The *T*_d,95%_ increased with the aromatic **VMA** content from 258 to 292 °C (Figure S16). DSC measurements showed that the *T*_g_ values of all the **VMA** copolymer samples were very close, ranging from 124 to 134 °C (Table 1, entries 5–7), seemingly increasing with *M_n_* rather than the **VMA** content. This may be due to a relatively small difference in *T*_g_ between the two polymers. Notably, the PMMA sample prepared by free‐radical polymerization showed a higher *T*_g_ (124 °C) than the commonly reported value of 105 °C. This has been reported previously and attributed to the syndiotactic‐rich character of the dyads in the polymer backbone.^[32]^

**Figure 2:**
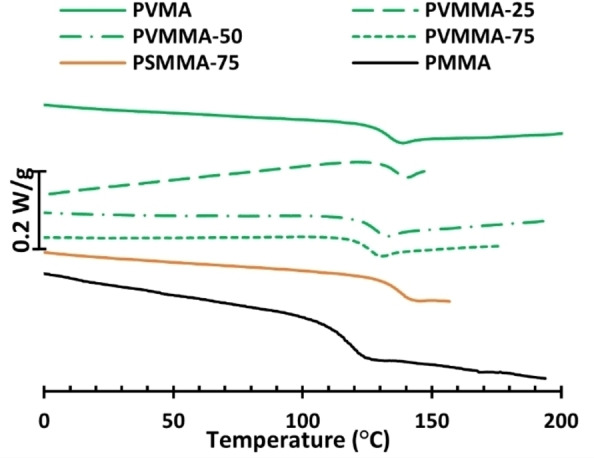
DSC thermograms of the homopolyesters PVMA, PMMA, and the copolymers of VMA and SMA with MMA.

As expected, at a given MMA content, the introduction of **SMA** units in the polymethacrylates had a more significant effect on *T*_g_ in comparison with **VMA**. Hence, the copolymer containing 25 mol% of **SMA** reached a *T*_g_ of 139 °C, while the corresponding copolymer with **VMA** displayed a *T*_g_ of 125 °C (Table 1, entries 7–8). Although bio‐based syringic acid is less available than vanillic acid, its effect on methacrylic materials is more promising for enhancing thermal properties (Figure 2).

### Rheological Properties

**Figure 3:**
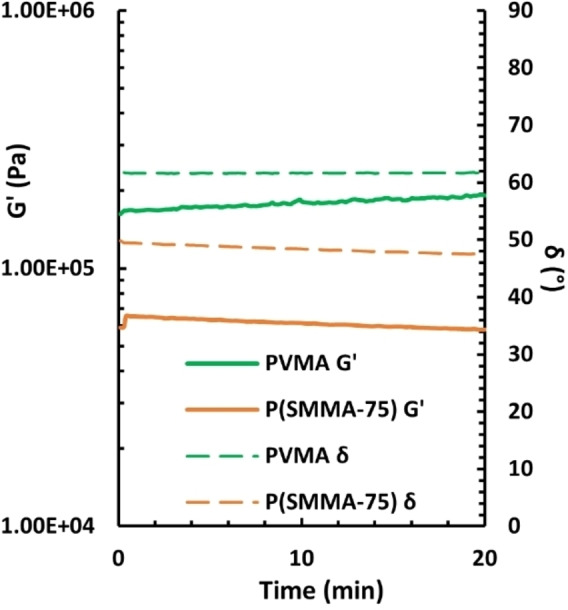
Variation of the melt shear storage modulus (G’) and phase shift (δ) during a time sweep of PVMA and PSMMA‐75 at 160 and 150 °C, respectively, measured at 1 Hz and 0.1 % strain.

The viscoelastic behavior of polymers is connected with molecular weight and molecular structure. Consequently, changes in the modulus and phase angle (*δ*) may indicate degradation by, e. g., polymer chain scission or crosslinking reactions.^[33]^ To verify that the polymethacrylate materials were thermally stable, the homopolymer **PVMA** and the copolymer **PSMMA‐75** were selected and studied by melt rheology in a plate−plate geometry. First, an isothermal experiment was performed on **PSMMA‐75** and **PVMA** at 150 or 160 °C, respectively, for 20 min (Figure 3). Although a slight increase in the shear storage modulus (G’) of **PVMA** was observed, the phase shift (*δ*) remained approximately constant during the analysis, indicating a high stability in the melt state. This implied that **PVMA** may be melt‐processed 30 °C above its *T*_g_ without significant degradation. Regarding **PSMMA‐75**, a slight decrease in both G’ and δ was observed, which may be attributed to an internal relaxation of the sample.

In addition, frequency sweeps of the **PVMA** and **PSMMA‐75** samples at increasing temperatures were performed. A time‐temperature superposition analysis of the data (Figures S17 and S18) showed that the master curves did not diverge significantly at higher temperatures, which indicated thermal stability of **PVMA** and **PSMMA‐75** up to 180 and 170 °C, respectively. In conclusion, these polymethacrylate samples appear to be melt‐processable, and thus promising for a wide range of applications.

### Life‐cycle Assessment and Toxicity

LCA of monomers **VMA** and **SMA** has been evaluated on three impact categories particularly relevant for biobased processes: global warming potential in 100‐years scale (GWP 100), terrestrial acidification and freshwater eutrophication. Such evaluation, and especially the proper comparison with commercial plastics, is difficult,^[34]^ since the synthesis of monomers **VMA** and **SMA** has been only developed on lab scale. On the other hand, a proper life‐cycle assessment at the early stage of the development enables to identify the hotspots directly and facilitates the process optimization towards lower impacts. Here we have applied our recently developed methodology, which integrates ex‐ante and prospective LCA and enables a more relevant comparison with commercial processes.^[35]^

Performing a conventional LCA of the two synthetic paths (i. e., anhydride path **‘anh**’ and acid chloride path ‘**acl**’) for **VMA** and **SMA** at the lab scale resulted in environmental impacts much higher than those of the fossil MMA (Figure S25, Lab‐scale). However, the application of ex‐ante LCA, which models the possible upscaled process, led to a reduction of the impacts, on average, by 91 % for global warming potential (a), 91 % for terrestrial acidification (b), and 89 % for freshwater eutrophication (c). The most significant reduction of impacts comes from the recovery of the solvents and other chemicals used in the process, which accounted for up to 89 % reduction of impacts compared to lab‐scale, indicated as Process synergies I in Figure S25. A sensitivity analysis was performed for three parameters: overall yield, recovery rate of solvents, and the amount of solvent used in synthetic steps (Figure S26, Tables S8 and S12). The most sensitive parameter was expectedly the recovery rate of solvents–e. g., a 15 % reduction of the considered recovery rate led to climate change impacts up to six times higher than the default scaling‐up values.

**Figure 4:**
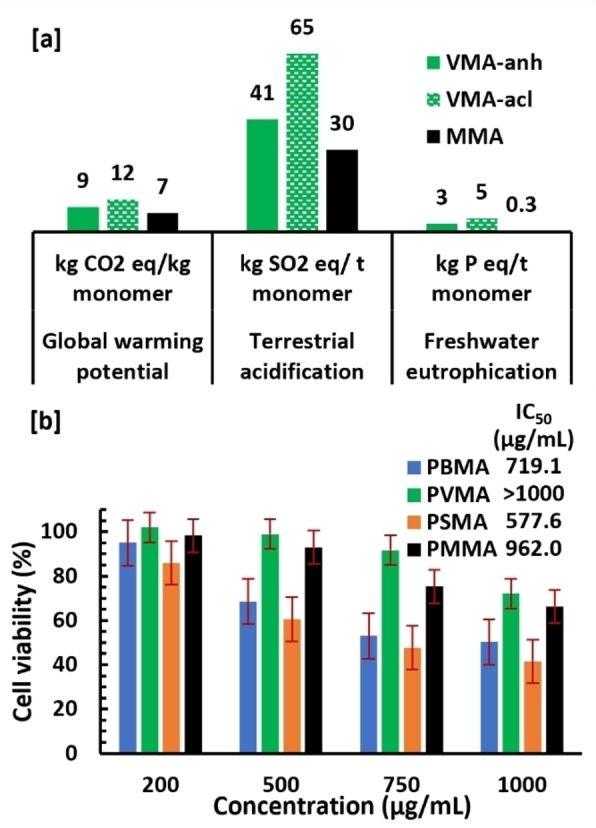
The environmental impacts of VMA using two different methods (VMA‐anh, VMA‐acl) compared to MMA [a], and cell viability of HeLa cells after exposure to the methacrylate polymers during 48 h [b].

Among the two monomers and different synthetic pathways evaluated, **VMA** via **anh** pathway has the lowest environmental impacts (Figure 4a). This is because vanillin is commercially produced at large scales^[36]^ and thus has ten times lower impacts compared to modeled syringic acid production, which still requires industrial maturity and optimization (Table S2). Although the acylation method using methacryloyl chloride has higher yields, it has no co‐products that can alleviate the environmental burdens of their production routes. Additionally, there is less room for theoretical yield improvement since the lab‐scale yield is already high and 2‐MeTHF has also higher environmental impacts than ethyl acetate (Table S11), contributing to the higher overall impact values.

Notably, the global warming and terrestrial acidification impacts of **VMA‐anh** (respectively 8 kg CO_2_eq/kg monomer and 44 kg SO_2_eq/t monomer) reach similar levels than fossil MMA (i. e., 7 kg CO_2_eq/kg monomer and 30 kg SO_2_eq/t monomer). It should be noted, that the production route of fossil MMA has been fine‐tuned for decades and this has resulted in an efficient process and low environmental impact that is hard to match. Full discussion of LCA evaluation including the data for monomer **SMA** and prospective LCA, is provided in SI.

Finally, the possible cytotoxicity of the methacrylate monomers and polymers was evaluated on HeLa cell line using MTT assay. The IC_50_ values of the monomers ranged from 0.17 to 0.34 mmol/L (Figure S27), which is comparable with reported results for other aromatic methacrylates.^[37]^ The polymers, however, showed significantly lower toxicity than monomers (Figure 4b). This was expected, since the polymers lack the reactive methacrylate group, which toxicity towards living organisms is known.^[38]^ At polymer concentrations of 200 μg/mL, the HeLa cells exhibited a high viability after 48 h (i. e., 86–100 %, Figure 4b). In contrast, in the case of the monomers, only 5–9 % of cells survived a shorter 24 h treatment at the same concentration (Figure S27). At higher concentrations, all the polymers resulted in a decrease in the cell viability (Figure 4b), and at 1000 μg/mL, 41–72 % of cells survived the 48 h treatment. This data is in the same range as a reference PMMA, which is routinely used in bio‐medical applications and is considered to have very low human toxicity,[^39^, ^40^] Consequently, **PBMA** and **PSMA** can be regarded as having low toxicity and **PVMA** can even be considered non‐toxic because its IC_50_ value is above 1000 μg/mL.^[41]^

## Conclusions

A straightforward chromatography‐free two‐step synthesis procedure was developed to obtain three methacrylate monomers from the lignin‐derived aromatic acids: syringic acid, vanillic acid, and 4‐hydroxybenzoic acid. These monomers were polymerized via free radical mechanism, yielding polymethacrylates with *T*_g_ values up to 197 °C and thermal stability up to about 300 °C. Copolymerizations with MMA enabled further tuning of *T*_g_ values and polymer properties. Rheological measurements indicated that the polymers were melt‐processable and thus potentially suitable as thermoplastics for various applications. A life‐cycle assessment of the monomers derived from vanillic and syringic acid indicated that, compared to conventional fossil‐based MMA, the potential effects on climate change and terrestrial acidification were similar after method optimization and scale‐up. Moreover, the toxicity of these polymethacrylates towards human HeLa cell lines was in the same range as PMMA. We envision that the accessible lignin‐based starting materials, straightforward synthetic procedures, and favorable thermal properties of the polymers, along with low toxicity and favorable LCA profile, will make these rigid bio‐based thermoplastics attractive for use in high‐performance applications.

## Experimental

Full details of materials, specific synthetic and polymerization procedures, structural characterization (NMR, HRMS, SEC), thermal characterization (TGA, DSC, rheology), LCA and cytotoxicity evaluation are provided in SI.

### General Synthetic Procedures

**Esterification of the benzoic acids**. The benzoic acid derivative (i. e., syringic, vanillic, or 4‐hydroxy acid) was dissolved in MeOH to the concentration of ca 25–50 mg/mL and heated to reflux. A catalytic amount of sulfuric acid was added, and the mixture was refluxed overnight (16 h). After that, the mixture was cooled and then concentrated under a vacuum. After adding ethyl acetate, the organic phase was washed with a saturated aq. NaHCO_3_ solution, dried on MgSO_4_, filtered, and then concentrated under vacuum. The obtained methyl ester was used without further purification in the next step (yields 92–97 %).

**Methacrylation using methacrylic anhydride**. The methyl ester was dissolved in ethyl acetate or 2‐MeTHF to the concentration of ca 50 mg/mL, followed by an addition of 1.5 equiv. of methacrylic anhydride and 0.02 equiv. of DMAP. The mixture was heated to 50 °C and stirred overnight (16 h). Next, the mixture was washed with 1 M aq. NaOH, 1 M aq. HCl and distilled water in sequence. The organic phase was dried over MgSO_4_, filtered, and concentrated under vacuum. The products were purified using column chromatography (EtOAc in petrol ether) with 48–73 % yields. Alternatively, **SMA** was obtained chromatography‐free in a 65 % yield after extraction, filtration and crystallization from EtOAc.

**Methacrylation using methacryloyl chloride**. The methyl ester was first dissolved in 2‐MeTHF (conc. ca 50 mg/mL) before adding 1.1 equiv. of Et_3_N. The flask was capped with a septum, flushed with argon, and cooled to 0 °C. Subsequently, 1.05 equiv. of methacryloyl chloride was slowly added dropwise, stirring the mixture overnight (16 h). Next, the crude product was washed with 1 M aq. NaOH, thrice with sat. aq. NaHCO_3_ and once with brine. The organic phase was dried over MgSO_4_, followed by drying under vacuum. The methacrylate monomer was obtained without further purification in 90, 92, and 93 % yields for **VMA**, **SMA**, and **BMA**, respectively.

**Free radical polymerization**. Approximately 500 mg of the monomer (i. e., **BMA**, **VMA**, or **SMA**) was dissolved in DMSO, EtOAc, 2‐MeTHF, CHCl_3_, GVL, or toluene to a concentration of ca 100 mg/mL before transfer into a polymerization vessel. Next, 0.5 mol% AIBN (0.1 mol% in case toluene was used) was added as a solution, and the mixture was degassed using argon for 1 h to remove the dissolved oxygen. The reactor was tightly sealed and placed in a preheated oven at 63 °C for 24 h. After cooling, the polymer was precipitated into an excess volume of methanol during stirring. Next, the polymer precipitate was filtered, collected, and dried under vacuum at 70–100 °C for 24 to 72 h until no mass change was observed.

Copolymerizations were performed using the same procedure as homopolymerizations, using 0.5 mol% AIBN relative to the MMA content. Copolymers were prepared from **VMA** or **SMA** and 25, 50, and 75 mol% MMA, respectively. After drying, the ratio obtained between the two components in the copolymers was determined from ^1^H NMR spectra. The copolymers were named after the lignin‐derived monomer with an additional “M,” symbolizing MMA, followed by the MMA content, e. g., **PVMMA‐75**.

## Conflict of Interests

The authors declare no conflict of interest.

1

## Supporting information

As a service to our authors and readers, this journal provides supporting information supplied by the authors. Such materials are peer reviewed and may be re‐organized for online delivery, but are not copy‐edited or typeset. Technical support issues arising from supporting information (other than missing files) should be addressed to the authors.

Supporting Information

## Data Availability

The data that support the findings of this study are available in the supplementary material of this article.
