# Supplementary material for: Aromatic Polymethacrylates from Lignin‐Based Feedstock: Synthesis, Thermal Properties, Life‐Cycle Assessment and Toxicity
Source: ChemSusChem. 2024 Oct 23;18(2):e202401239. doi: 10.1002/cssc.202401239 (PMC11739823; doi:10.1002/cssc.202401239)
Supplement: Supplementary file 1 — Supporting Information [file CSSC-18-e202401239-s001.pdf]

ChemSusChem

Supporting Information

Aromatic Polymethacrylates from Lignin-Based Feedstock: Synthesis, Thermal Properties, Life-Cycle Assessment and Toxicity

Rauno Sedrik, Olivier Bonjour, Nariê Rinke Dias de Souza, Alina Ismagilova, Iris Tamsalu, Veljo Kisand, Francesco Cherubini, Patric Jannasch,* and Lauri Vares*

Aromatic Polymethacrylates from Lignin-Based Feedstock: Synthesis, Thermal Properties, Toxicity and Life-Cycle Assessment

Rauno Sedrik[1], Olivier Bonjour[2], Iris Tamsalu[1], Nariê Rinke Dias de Souza[3], Alina Ismagilova[1], Veljo Kisand[1], Francesco Cherubini[3], Patric Jannasch[2], Lauri Vares[1]

[1]Institute of Technology, University of Tartu, Nooruse 1, Tartu 50411, Estonia

[2]Department of Chemistry, Lund University, Box 124, Lund 221 00, Sweden

[3]Industrial Ecology Programme, Department of Energy and Process Engineering, Norwegian University of Science and Technology, Trondheim, Norway

Table of contents

Materials and methods 2

Structural analysis 3

Thermal properties 3

Cytotoxicity evaluation using HeLa cells 4

Experimental procedures 5

Monomer synthesis 5

Synthesis of vanillic acid methyl ester methacrylate VMA 5

Synthesis of 4-hydroxybenzoic acid methyl ester methacrylate BMA 6

Synthesis of syringic acid methyl ester methacrylate SMA 7

Free radical polymerization 8

Polymerization of PMMA 8

Polymerization of PBMA 8

Polymerization of PVMA 9

Polymerization of PSMA 9

Copolymerization of PVMMA-25 9

Copolymerization of PVMMA-50 9
Copolymerization of PVMMA-75 10
Copolymerization of PSMMA-75 10
NMR spectra 11
Monomer ^{1}H and ^{13}C spectra. 11
Polymer ^{1}H NMR spectra 14
Polymer characterization 18
Thermal degradation 18
Rheology 19
SEC curves 21
Life cycle assessment and toxicity 21
General LCA framework 21
Phase I: Goal and scope definition 24
Phase II: Scaling up foreground data 26
Sensitivity analysis 28
Phase III: Projection of background data 29
Results 33
Visualization of results 35
Cell viability of monomers 37
References 37

Materials and methods

4-Hydroxybenzoic acid (99%) and vanillic acid (97%) were obtained from Acros Organics. Syringic acid (98%) and methacrylic anhydride (MAA, 94%) were obtained from Alfa Aesar, while methacrylic acid (99%) was obtained from Thermo Scientific. Methanol (99.8%), ethyl acetate (99%), chloroform (99.8%), and methyl methacrylate (MMA, 99%) were obtained from Fisher Chemicals. Dimethyl sulfoxide (DMSO, 99.9%), 2-methyltetrahydrofuran (2-MeTHF, 99.5%), and methacryloyl chloride (97%) were obtained from Sigma Aldrich. All solvents and

reagents were used as received. Silica gel 60 (0.040−0.063 mm, 230−400 mesh) was used for flash chromatography. 3-(4,5-Dimethyl-2-thiazolyl)-2,5-diphenyl-2H-tetrazorium bromide (MTT) from Sigma Aldrich was used for the cytotoxicity tests.

Structural analysis

The structures of the monomers and polymers were characterized by nuclear magnetic resonance (NMR) spectroscopy using a 400 MHz Bruker spectrometer. The samples were measured in chloroform-*d*, and the ^{1}H and ^{13}C spectra were recorded at 400 and 101 MHz, respectively. Residual solvent signals were used for calibration (7.26 ppm and 77.16 ppm for $CDCl_3$).

A hybrid instrument consisting of a Varian 910 FT-ICR-MS and a Varian J-320 3Q mass spectrometer with a horizontal 7 T superconductive magnet and a nanoESI source was used for HRMS analysis. Varian Omega 9.1.21 software was used for the experiment setup and data acquisition.

The molecular weights of the polymers were determined by size-exclusion chromatography (SEC) using THF as an eluent. A Shimadzu Prominence setup was used with a refractive index detector (RID-20A) and three Shodex columns (KF-805, -804, and -802.5, coupled in series). All samples were run at 40 °C at an elution rate of 1 mL/min. Polystyrene standards (M_n = 3.18, 10.44, 28.77, 74.80, 230.9, 473.6 kg/mol) were used for calibration, and the results were analyzed by Shimadzu LabSolution software.

Thermal properties

Thermogravimetric analysis (TGA) was performed using TA Instruments TGA Q500 apparatus to determine the thermal stability of the polymers under an N_2 flux of 60 mL/min. Samples of 2.2–13.7 mg were kept isothermally at 120–150 °C for up to 60 min to remove solvent residues. After equilibration at 30–50 °C, the temperature was increased to 600 °C at a heating rate of 10 °C/min. The thermal decomposition temperature ($T_{d,95\%}$) was determined at 5% weight loss.

Differential scanning calorimetry (DSC) was carried out using a TA Instruments DSC Q2000 differential scanning calorimeter. Dried samples of 2.6–8.3 mg were transferred to aluminum pans and sealed. The samples were first heated to 150–250 °C, depending on their respective onset of decomposition, at a rate of 10 °C/min. After an isothermal period of 5 min, the samples were cooled to -50 °C followed by a 5 min isothermal period. Finally, the samples were heated

to the original temperature at 10 °C/min. T_g values were evaluated from the thermograms as the middle point between the onset and offset temperatures of the glass transitions.

Dynamic rheology measurements were performed with a TA Instruments Advanced Rheometer AR2000 ETC. The experiments were carried out using parallel plates (Ø = 15 mm). Disks of **PVMA** and **PSMMA-75** samples (Ø = 15 mm, t = 1 mm) were hot-pressed in a steel mold placed between two aluminum plates using a hydraulic press (Specac, GS15011) at 160 °C for 2 min. The sample was then cooled to room temperature for 5 min. Time sweeps were carried out at 150 or 160 °C for **PSMMA-75** and **PVMA**, respectively, during 20 min at 1 Hz and 0.1% strain, which was within the linear viscoelastic region. In addition, frequency sweeps from 0.01 to 100 Hz were performed at a strain of 0.1%, with the temperature increasing from 150 to 170 °C for **PSMMA-75** and from 160 to 180 °C for **PVMA**.

Cytotoxicity evaluation using HeLa cells

The cytotoxicity of the monomers and polymers was evaluated towards human carcinoma HeLa cells using an MTT assay.[1] We followed procedures previously described in literature after some modification.[2,3]

Human cervical carcinoma HeLa cells were plated to the 96-well plates at 5000 cells/well in 100 μL Dulbecco's Modified Eagle Medium (DMEM) with 1% penicillin-streptomycin and 10% fetal bovine serum (FBS) and was then incubated at 37 °C in 5% CO_2 and 100% relative humidity. After 24 h incubation, to ensure adhesion for cell cytotoxicity assay, 100 μL of sample solutions in DMEM were added. A culture medium without additives was used as a negative control, and a medium with 2% H_2O_2 was used as a positive control. The samples were dissolved in DMSO at the concentration of 50 mg/mL. These sample solutions were diluted with the culture medium to concentrations of 20, 40, 100, and 200 µg/mL for monomers and 200, 500, 750, and 1000 µg/mL for polymers. After the cells were exposed to the monomers and polymers for 24 and 48 h respectively, the medium was removed. Next, 100 μL of fresh culture medium and 20 μL of MTT dissolved in phosphate-buffered saline (PBS) solution at a concentration of 5 mg/mL were added to each well. After 2 h of incubation, the medium was removed, and 200 μL of DMSO was added to each well to re-suspend intracellularly stored MTT formazan. The optical density of the formazan product was measured at 560 nm (OD_{560}). All samples were tested 5 times to ensure test reproducibility. Relative cell viability was calculated using the following equation: relative cell viability (% of

control) = (OD_{560} treated wells/OD_{560} control) × 100. The IC_{50} values of materials were calculated using ED50plus v1.0 software.

Experimental procedures

Monomer synthesis

Synthesis of vanillic acid methyl ester methacrylate VMA

Vanillic acid (1.9 g, 11.3 mmol) was dissolved in 15 ml of methanol. The mixture was heated to reflux, 2 drops of H_2SO_4 were added, and the mixture was refluxed overnight (16 h). The mixture was concentrated under vacuum, 10 ml of ethyl acetate was added, and the mixture was neutralized with saturated aq. $NaHCO_3$ solution. The product was dried and used without any further purification in the next step (yield 92%).

Vanillic acid methyl ester (1.01 g, 5.5 mmol) obtained in the previous step was dissolved in ethyl acetate and 1.5 equiv. of methacrylic anhydride (1.3 ml) and 0.02 equiv. of DMAP (13 mg, mmol) were added. The mixture was heated to 50 °C and stirred overnight (16 h). Next, the mixture was consequently washed with 1 M NaOH, 1 M HCl, and distilled water, dried on $MgSO_4$, and purified using flash chromatography (15% EtOAc in petrol ether). After drying, the pure monomer was obtained as a viscous liquid (0.69 g, 50% yield).

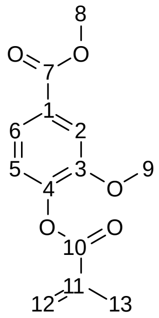

^{1}H NMR (400 MHz, $CDCl_3$): δ 7.64 (dd $^2J_{HH}$ = 8.2 Hz, $^4J_{HH}$ =1.8 Hz, $CH_{(2,6)}$, 2H), 7.10 (d $^2J_{HH}$ = 8.0 Hz, $CH_{(5)}$, 1H), 6.33 (m, $CH_{2(12E)}$, 1H), 5.73 (m, $CH_{2(12Z)}$, 1H), 3.86 (s, $CH_{3(8)}$, 3H), 3.82 (s, $CH_{3(9)}$, 3H), 2.02 (s, $CH_{3(13)}$, 3H). **(Figure S3)**

^{13}C NMR (100 MHz, $CDCl_3$): 166.3 ($C{=}O_{(7)}$), 164.8 ($C{=}O_{(10)}$), 151.1 ($C_{(3)}$), 143.8 ($C_{(4)}$), 135.3 ($C_{(11)}$), 128.6 ($C_{(1)}$), 127.5 ($CH_{2(12)}$), 122.8 ($CH_{(6)}$), 122.5 ($CH_{(2)}$), 113.4 ($CH_{(5)}$), 56.0 ($CH_{3(9)}$), 52.1 ($CH_{3(8)}$), 18.2 ($CH_{3(13)}$). **(Figure S4)**

HRMS (nESI): calculated for $C_{13}H_{14}O_5$ $[M + Na]^+$ 273.07334, found 273.0734.

R_f: 0.40 (15% EtOAc in petrol ether)

Chromatography-free alternative method for the second step

Vanillic acid methyl ester (1.02 g, 5.6 mmol) was dissolved in 20 ml of 2-MeTHF, and 1.1 equiv. (0.93 ml) of triethylamine was added to the mixture. The flask was capped with a septum, flushed with argon, and cooled to 0 °C. Thereafter 1.05 equiv. of freshly distilled methacryloyl chloride (0.58 ml) was slowly added to the reaction and the mixture was stirred overnight for 16 hours. Next, the crude product was washed consequently with 20 ml of 1 M

NaOH, 3 times with 20 ml of saturated $NaHCO_3$ and lastly with 20 ml of brine. The organic phase was collected and dried over $MgSO_4$, followed by drying under vacuum. The product was obtained as a viscous liquid (1.24 mg, yield 90%).

Synthesis of 4-hydroxybenzoic acid methyl ester methacrylate BMA

4-hydroxybenzoic acid (1.16 g, 8.4 mmol) was dissolved in 10 ml of methanol. The mixture was heated to reflux and a drop of H_2SO_4 was added, and the mixture was refluxed overnight (16 h). The mixture was concentrated under vacuum, 15 ml of ethyl acetate was added, and the mixture was neutralized with saturated aq. $NaHCO_3$ solution. The product was dried and used without any further purification in the next step (yield 93%).

4-hydroxybenzoic acid methyl ether (1.05 g, 6.9 mmol) obtained in the previous step was dissolved in ethyl acetate and 1.5 equiv. of methacrylic anhydride (1.5 ml) and 0.02 equiv. of DMAP (16 mg) were added. The mixture was heated to 50 °C and stirred overnight (16 h). Next, the mixture was washed with 1 M aq. NaOH, 1 M aq. HCl and distilled water consequently. The organic phase was dried on $MgSO_4$ and purified using flash chromatography (15% EtOAc in petrol ether). After drying, the pure monomer was obtained as a white powder (0.73 g, 48% yield).

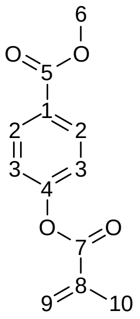

^{1}H NMR (400 MHz, $CDCl_3$): δ 8.09 (d, $^2J_{HH}$ = 8.7 Hz, $CH_{(2)}$, 2H), 7.22 (d, $^2J_{HH}$ = 8.7 Hz, $CH_{(3)}$, 2H), 6.37 (m, $CH_{2(9E)}$, 1H), 5.79 (m, $CH_{2(9Z)}$, 1H), 3.92 (s, $CH_{3(6)}$, 3H), 2.06 (s, $CH_{3(10)}$, 3H). **(Figure S1)**

^{13}C NMR (100 MHz, $CDCl_3$): 166.4 (C=$O_{(5)}$), 165.3 (C=$O_{(7)}$), 154.7 ($C_{(4)}$), 135.6 ($C_{(8)}$), 131.2 ($CH_{(2)}$), 127.9 ($CH_{2(9)}$), 127.7 ($C_{(1)}$), 121.7 ($CH_{(3)}$), 52.3 ($CH_{3(6)}$), 18.4 ($CH_{3(10)}$). **(Figure S2)**

HRMS (nESI): calculated for $C_{12}H_{12}O_4$ $[M + Na]^+$ 243.06278, found 243.0628.

R_f: 0.46 (15% EtOAc in petrol ether)

Chromatography-free alternative method for the second step

4-hydroxybenzoic acid methyl ester (1.04 g, 6.84 mmol) was dissolved in 20 ml of 2-MeTHF, and 1.1 equiv. (1.0 ml) of triethylamine was added to the mixture. The flask was capped with a septum, flushed with argon, and cooled to 0 °C. Thereafter 1.05 equiv. of freshly distilled methacryloyl chloride (0.7 ml) was slowly added to the reaction and the mixture was stirred overnight for 16 hours. Next, the crude product was washed consequently with 20 ml of 1 M NaOH, 3 times with 20 ml of saturated $NaHCO_3$ and lastly with 25 ml of brine. The organic

phase was collected and dried over $MgSO_4$, followed by drying under vacuum. The product was obtained as a white powder (1.406 mg, yield 93%).

Synthesis of syringic acid methyl ester methacrylate SMA

Syringic acid (5.24 g, 26.4 mmol) was dissolved in 100 ml of methanol. The mixture was heated to reflux, 20 drops of H_2SO_4 were added, and the mixture was refluxed overnight (16 h). The mixture was concentrated under vacuum, 20 ml of ethyl acetate was added, and the mixture was neutralized with saturated aq. $NaHCO_3$ solution. The product was dried and used without any further purification in the next step (yield 97%).

Syringic acid methyl ester (1.53 g, 7.1 mmol) obtained in the previous step was dissolved in ethyl acetate and 1 equiv. of methacrylic anhydride (1.1 ml) and 0.02 equiv. of DMAP (mg, mmol) were added. The mixture was heated to 50 °C and stirred overnight (16 h). Next, the mixture was washed with brine, dried on $MgSO_4$, and passed through a thin layer of silica. Afterward, the product was recrystallized in ethyl acetate and washed with petrol ether. After drying, the pure monomer was obtained as a white powder (1.306 g, yield 65%).

Alternative chromatographic purification:

The crude reaction mixture was consequently washed with 1 M NaOH, 1 M HCl, and distilled water, dried on $MgSO_4$, and purified using flash chromatography (15% EtOAc in petrol ether). After drying, the pure monomer was obtained as a white powder (yield 73%).

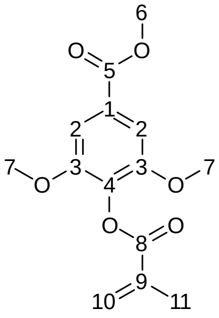

^{1}H NMR (400 MHz, $CDCl_3$): δ 7.33 (s, $CH_{(2)}$, 2H), 6.40 (m, $CH_{2(10E)}$, 1H), 5.78 (m, $CH_{2(10Z)}$, 1H), 3.92 (s, $CH_{3(6)}$, 3H), 3.86 (s, $CH_{3(7)}$, 6H), 2.08 (s, $CH_{3(11)}$, 3H). **(Figure S5)**

^{13}C NMR (100 MHz, $CDCl_3$): 166.5 (C=$O_{(5)}$), 164.8 (C=$O_{(8)}$), 152.3 ($C_{(3)}$), 135.3 ($C_{(9)}$), 132.9 ($C_{(4)}$), 128.0 ($C_{(1)}$), 127.7 ($CH_{2(10)}$), 106.4 ($CH_{(2)}$), 56.4 ($CH_{3(7)}$), 52.4 ($CH_{3(6)}$), 18.5 ($CH_{3(11)}$). **(Figure S6)**

HRMS (nESI): calculated for $C_{14}H_{16}O_6$ $[M + Na]^+$ 303.08391, found 303.0839.

R_f: 0.37 (20% EtOAc in petrol ether)

Chromatography-free alternative method for the second step

Syringic acid methyl ester (928 mg, 4.37 mmol) was dissolved in 20 ml of 2-MeTHF, and 1.1 equiv. (0.67 ml) of triethylamine was added to the mixture. The flask was capped with a septum, flushed with argon, and cooled to 0 °C. Thereafter 1.05 equiv. of freshly distilled

methacryloyl chloride (0.45 ml) was slowly added to the reaction and the mixture was stirred overnight for 16 hours. Next, the crude product was washed consequently with 20 ml of 1 M NaOH, 3 times with 20 ml of saturated $NaHCO_3$ and lastly with 25 ml of brine. The organic phase was collected and dried over $MgSO_4$, followed by drying under vacuum. The product was obtained as a white powder (1.127 g, yield 92%).

Free radical polymerization

Table S1. Polymerization optimization in different solvents.

Entry	Polymer	Solvent	M_n[a] (kg/mol)	$Đ$[a]	Isolated yield (%)
1	PBMA	DMSO	62	3.6	71
2	PBMA	GVL	14	2.1	64
3	PBMA	Toluene	19	2.3	67
4	PBMA	EtOAc	48	2.4	45
5	PVMA	DMSO	53	3.8	81
6	PVMA	Toluene	8	1.8	40
7	PVMA	Toluene[b]	38	2.9	47
8	PVMA	Chloroform	36	2.5	50
9	PVMA	EtOAc	48	2.6	57
10	PVMA	GVL	48	3.7	87
11	PVMA	2-MeTHF	15	2.5	76
12	PSMA	DMSO	53	4.1	67
13	PSMA	GVL	94	2.1	59
14	PSMA	EtOAc	15	2.1	31
15	PSMA	Chloroform	50	1.9	55
16	PSMA	Cyrene	34	1.7	53

[a]Measured by SEC in THF. [b]0.1 mol% of AIBN used.

Polymerization of PMMA

MMA (289 mg, 2.88 mmol) was dissolved in GVL (2.9 ml), and 0.5 mol% AIBN (0.55 mg, 0.003 mmol) was added as a solution in DMSO. The mixture was degassed with argon for 1 hour to reduce the amount of inhibiting oxygen in the solvent. The polymerization tube was sealed, and the polymerization was carried out at 63 °C for 24 h. After that, the polymer was precipitated into 300 ml MeOH while stirring. Next, the precipitate was filtered, washed with methanol, collected, and dried under a vacuum at 80 °C. After drying, the white powdery polymer weighed 206 mg (yield 71 %).

Polymerization of PBMA

BMA (1116 mg, 5.06 mmol) was dissolved in DMSO (11.1 ml), and 0.5 mol% AIBN (4.1 mg, 0.02 mmol) was added as a solution in DMSO. The mixture was degassed with argon for 1

hour to reduce the amount of inhibiting oxygen in the solvent. The polymerization tube was sealed, and the polymerization was carried out at 63 °C for 24 h. After that, the polymer was precipitated into 800 ml MeOH while stirring. Next, the precipitate was filtered, washed with methanol, collected, and dried under a vacuum at 80 °C. After drying, the white powdery polymer weighed 863 mg (yield 77 %).

Polymerization of PVMA

VMA (1301 mg, 5.19 mmol) was dissolved in DMSO (13.0 ml), and 0.5 mol% AIBN (4.2 mg, 0.026 mmol) was added as a solution in DMSO. The mixture was degassed with argon for 1 hour to reduce the amount of inhibiting oxygen in the solvent. The polymerization tube was sealed, and the polymerization was carried out at 63 °C for 24 h. After that, the polymer was precipitated into 400 ml MeOH while stirring. Next, the precipitate was filtered, washed with methanol, collected, and dried under a vacuum at 80 °C. After drying, the white powdery polymer weighed 1095 mg (yield 84 %).

Polymerization of PSMA

SMA (98 mg, 0.35 mmol) was dissolved in DMSO (2.0 ml), and 0.5 mol% AIBN (0.3 mg, 0.002 mmol) was added as a solution in DMSO. The mixture was degassed with argon for 1 hour to reduce the amount of inhibiting oxygen in the solvent. The polymerization tube was sealed, and the polymerization was carried out at 63 °C for 24 h. After that, the polymer was precipitated into 100 ml MeOH while stirring. Next, the precipitate was filtered, washed with methanol, collected, and dried under a vacuum at 80 °C. After drying, the white powdery polymer weighed 44 mg (yield 46 %).

Copolymerization of PVMMA-25

VMA (164 mg, 0.65 mmol) and MMA (20 mg, 0.21 mmol) were dissolved in DMSO (1.8 ml), and 0.5 mol% AIBN (0.1 mg, 0.001 mmol) was added as a solution in DMSO. The mixture was degassed with argon for 1 hour to reduce the amount of inhibiting oxygen in the solvent. The polymerization tube was sealed, and the polymerization was carried out at 63 °C for 24 h. After that, the polymer was precipitated into 100 ml MeOH while stirring. Next, the precipitate was filtered, washed with methanol, collected, and dried under a vacuum at 70 °C. After drying, the white powdery polymer weighed 169 mg (yield 91 %).

Copolymerization of PVMMA-50

VMA (141 mg, 0.56 mmol) and MMA (60 mg, 0.56 mmol) were dissolved in DMSO (2.0 ml), and 0.5 mol% AIBN (0.4 mg, 0.003 mmol) was added as a solution in DMSO. The mixture

was degassed with argon for 1 hour to reduce the amount of inhibiting oxygen in the solvent. The polymerization tube was sealed, and the polymerization was carried out at 63 °C for 24 h. After that, the polymer was precipitated into 100 ml MeOH while stirring. Next, the precipitate was filtered, washed with methanol, collected, and dried under a vacuum at 70 °C. After drying, the white powdery polymer weighed 157 mg (yield 78 %).

Copolymerization of PVMMA-75

VMA (424 mg, 1.69 mmol) and MMA (580 mg, 5.08 mmol) were dissolved in DMSO (10.0 ml), and 0.5 mol% AIBN (4.1 mg, 0.025 mmol) was added as a solution in DMSO. The mixture was degassed with argon for 1 hour to reduce the amount of inhibiting oxygen in the solvent. The polymerization tube was sealed, and the polymerization was carried out at 63 °C for 24 h. After that, the polymer was precipitated into 400 ml MeOH while stirring. Next, the precipitate was filtered, washed with methanol, collected, and dried under a vacuum at 70 °C. After drying, the white powdery polymer weighed 793 mg (yield 79 %).

Copolymerization of PSMMA-75

SMA (1019 mg, 3.63 mmol) and MMA (1091 mg, 10.90 mmol) were dissolved in DMSO (21.1 ml), and 0.5 mol% AIBN (8.9 mg, 0.055 mmol) was added as a solution in DMSO. The mixture was degassed with argon for 1 hour to reduce the amount of inhibiting oxygen in the solvent. The polymerization tube was sealed, and the polymerization was carried out at 63 °C for 24 h. After that, the polymer was precipitated into 1000 ml MeOH while stirring. Next, the precipitate was filtered, washed with methanol, collected, and dried under a vacuum at 70 °C. After drying, the white powdery polymer weighed 1.744 mg (yield 83 %).

NMR spectra

Monomer ^{1}H and ^{13}C spectra.

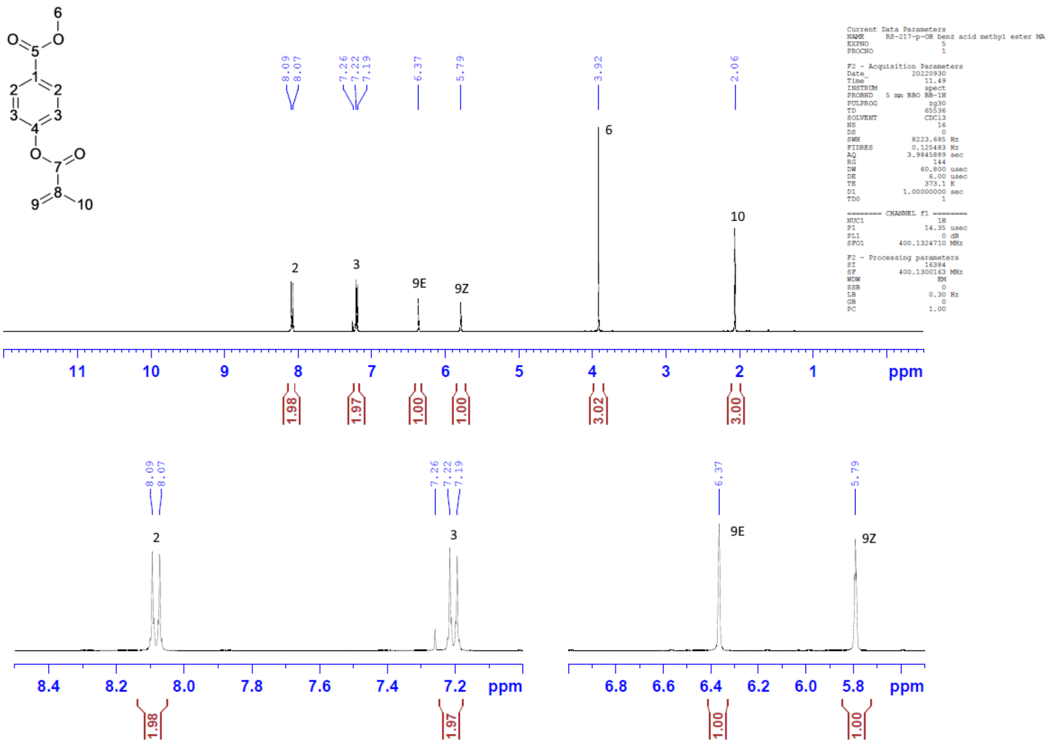

Figure S1. ^{1}H spectrum of BMA measured in $CDCl_3$.

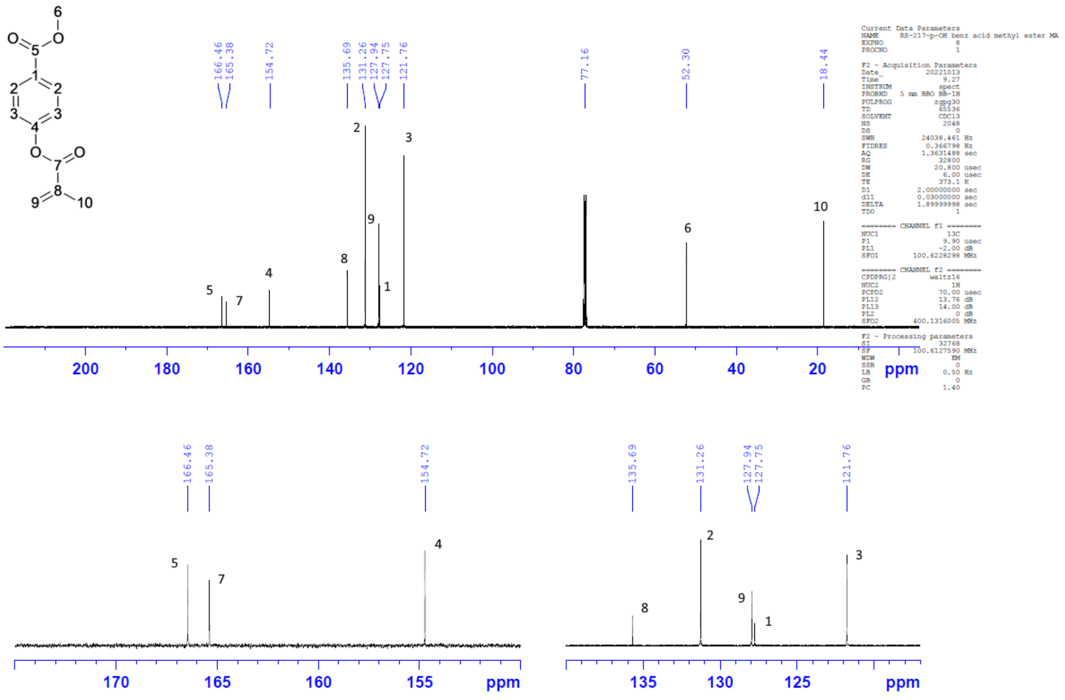

Figure S2. ^{13}C spectrum of BMA measured in $CDCl_3$.

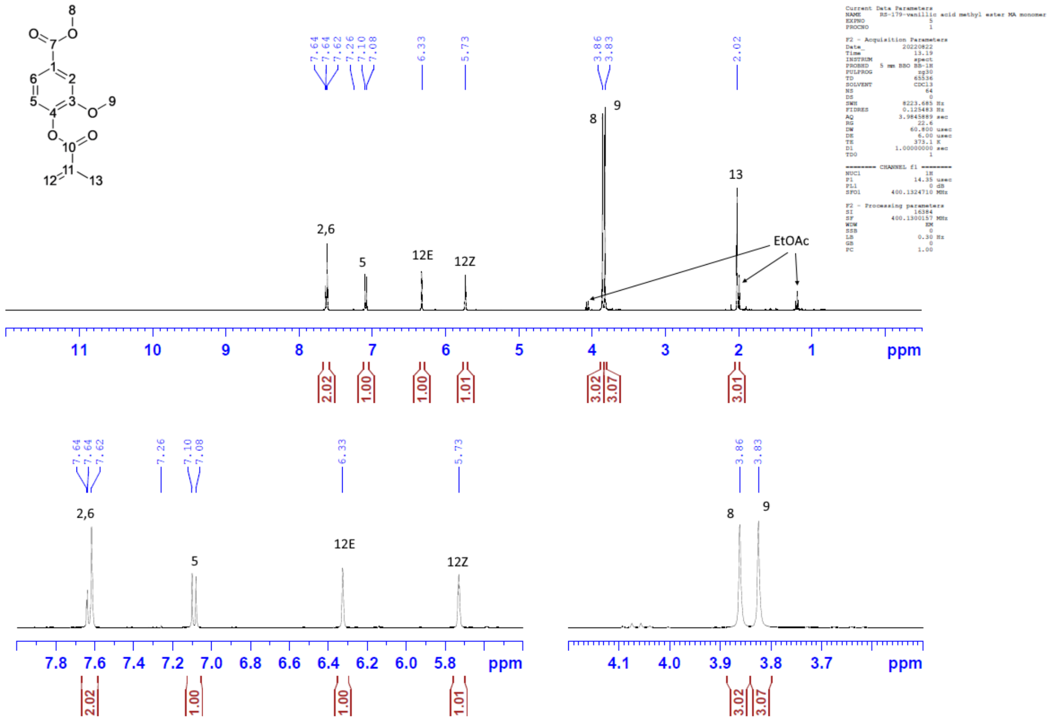

Figure S3. ^{1}H spectrum of VMA measured in $CDCl_3$.

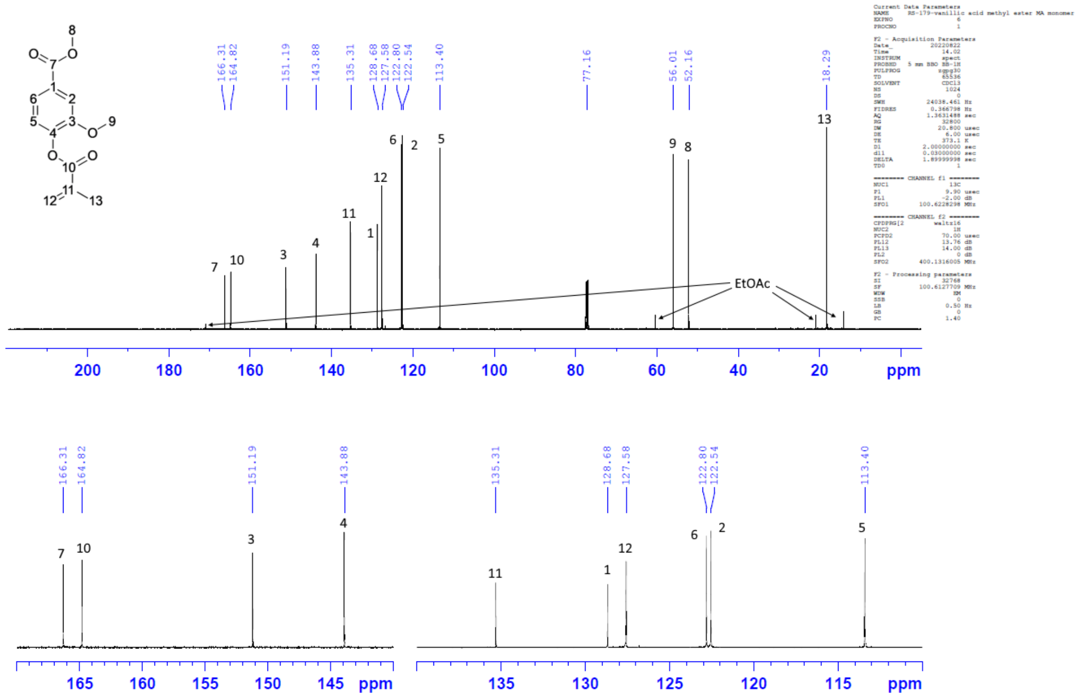

Figure S4. ^{13}C spectrum of VMA measured in $CDCl_3$.

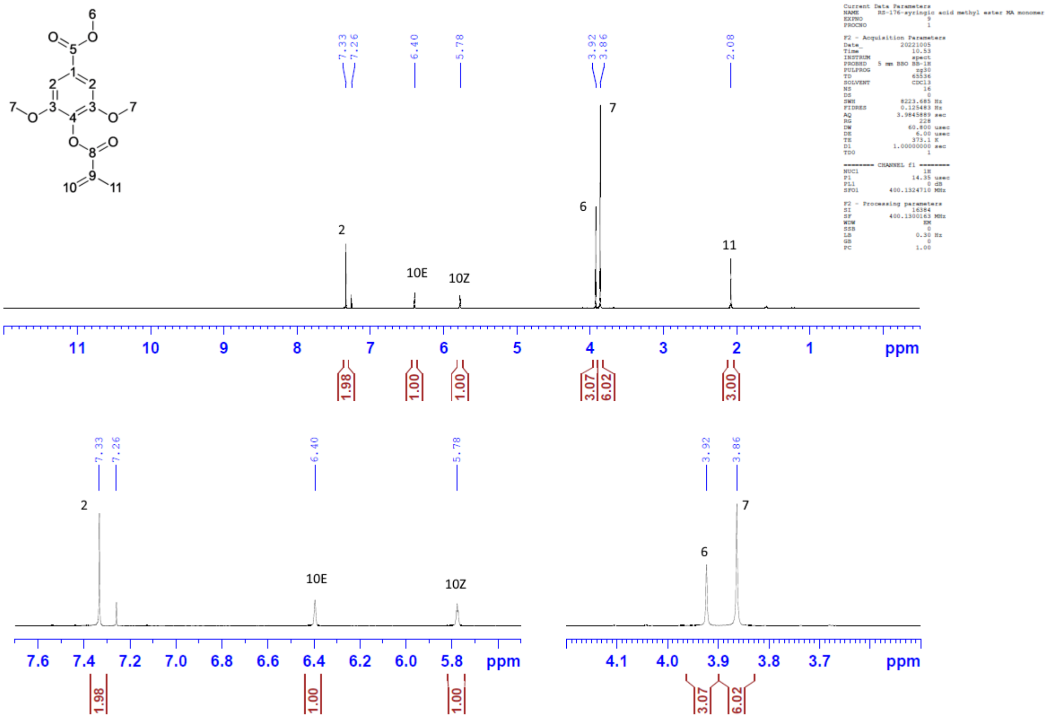

Figure S5. ^{1}H spectrum of SMA measured in $CDCl_3$.

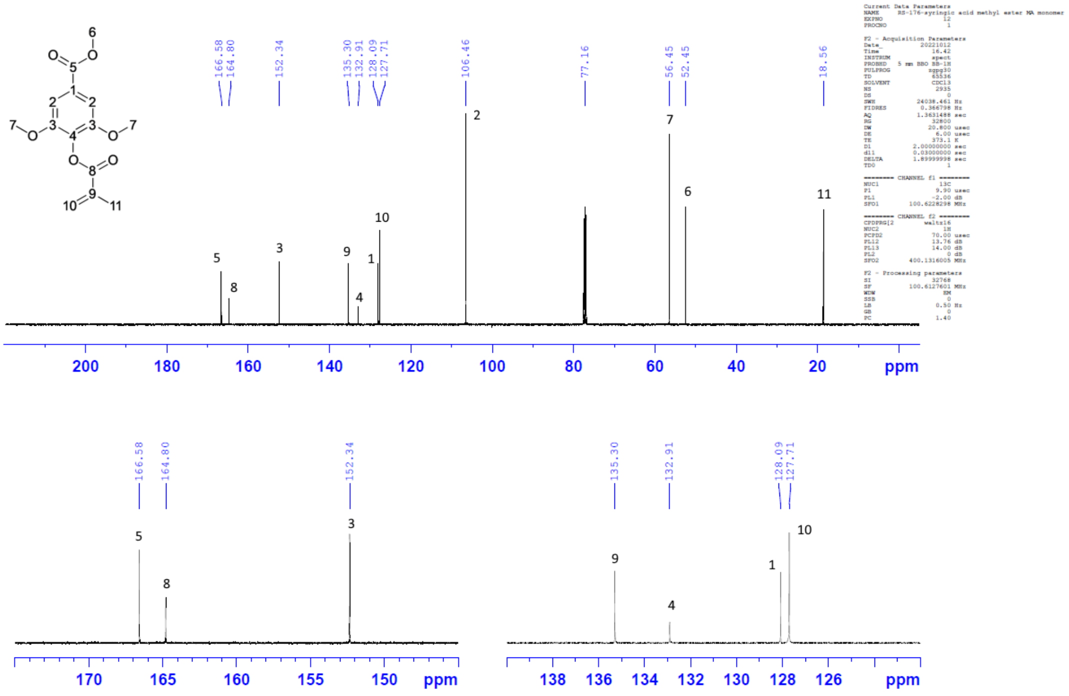

Figure S6. ^{13}C spectrum of SMA measured in $CDCl_3$.

Polymer ^{1}H NMR spectra

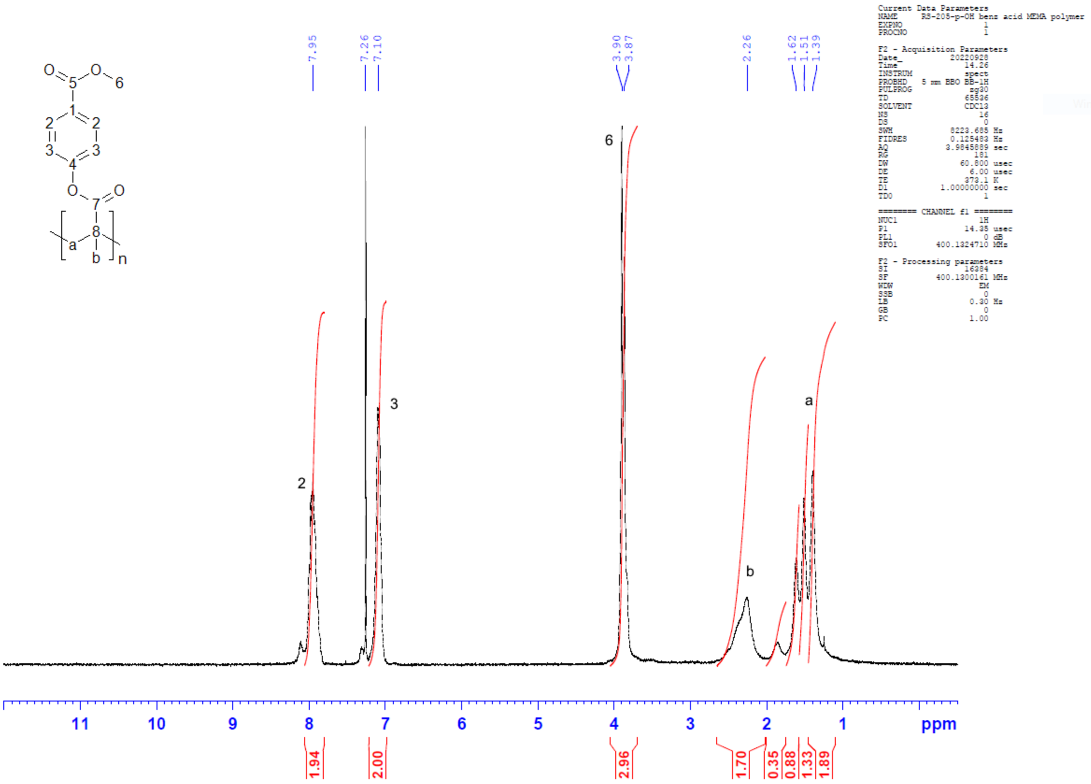

Figure S7. ^{1}H spectrum of PBMA measured in $CDCl_3$.

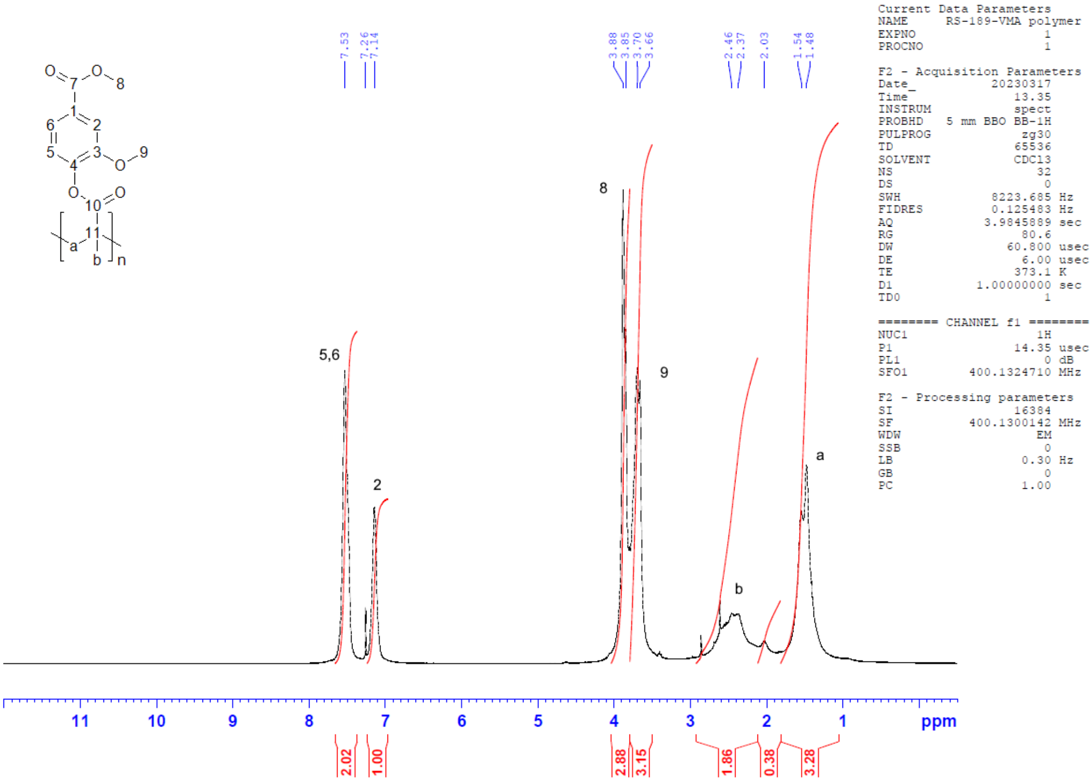

Figure S8. ^{1}H spectrum of PVMA measured in $CDCl_3$.

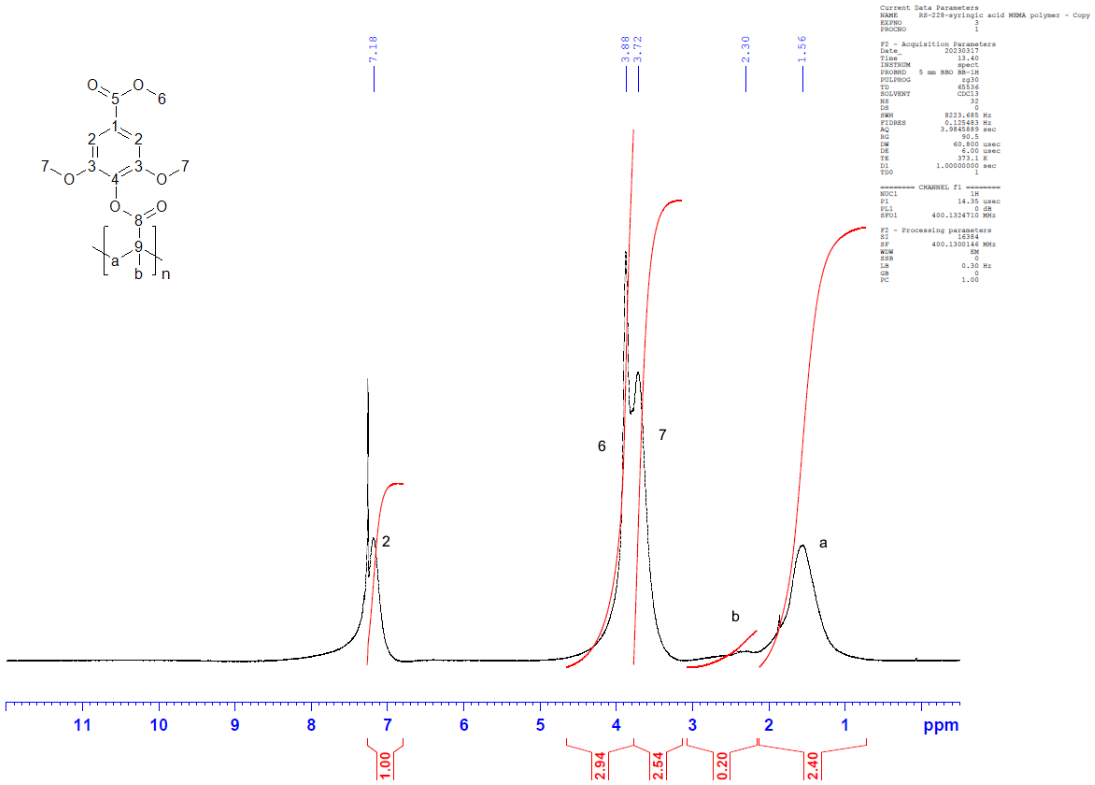

Figure S9. ^{1}H spectrum of PSMA measured in $CDCl_3$.

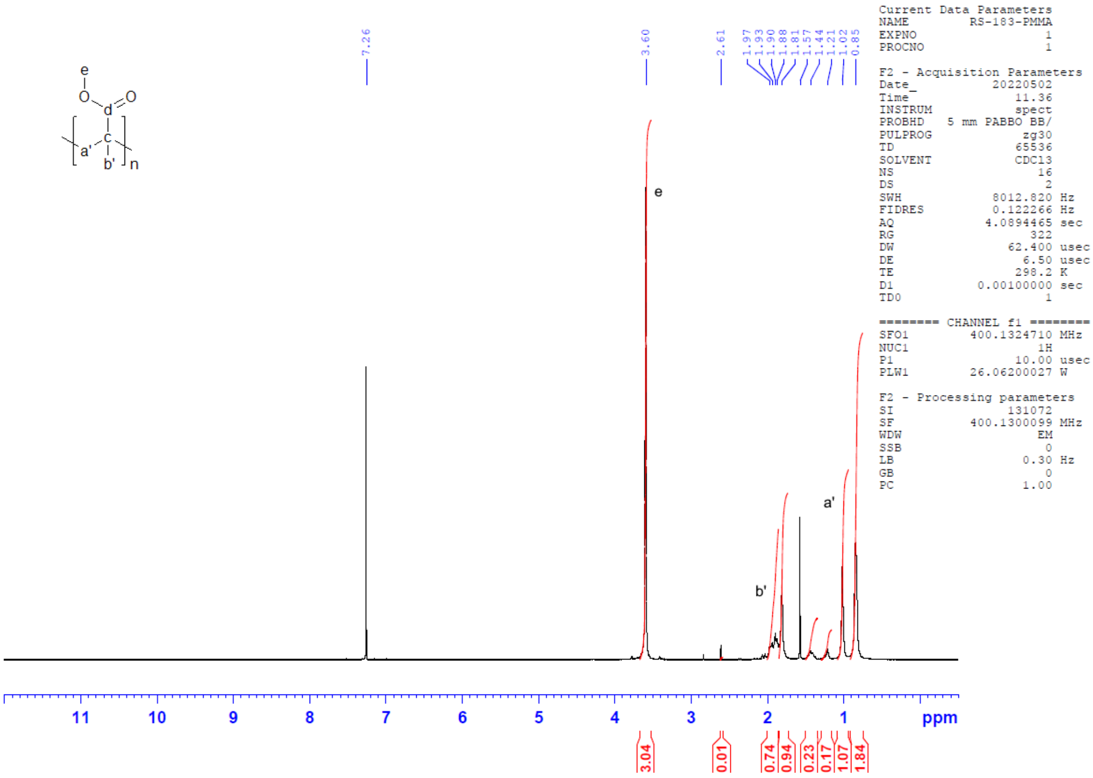

Figure S10. ^{1}H spectrum of PMMA measured in $CDCl_3$.

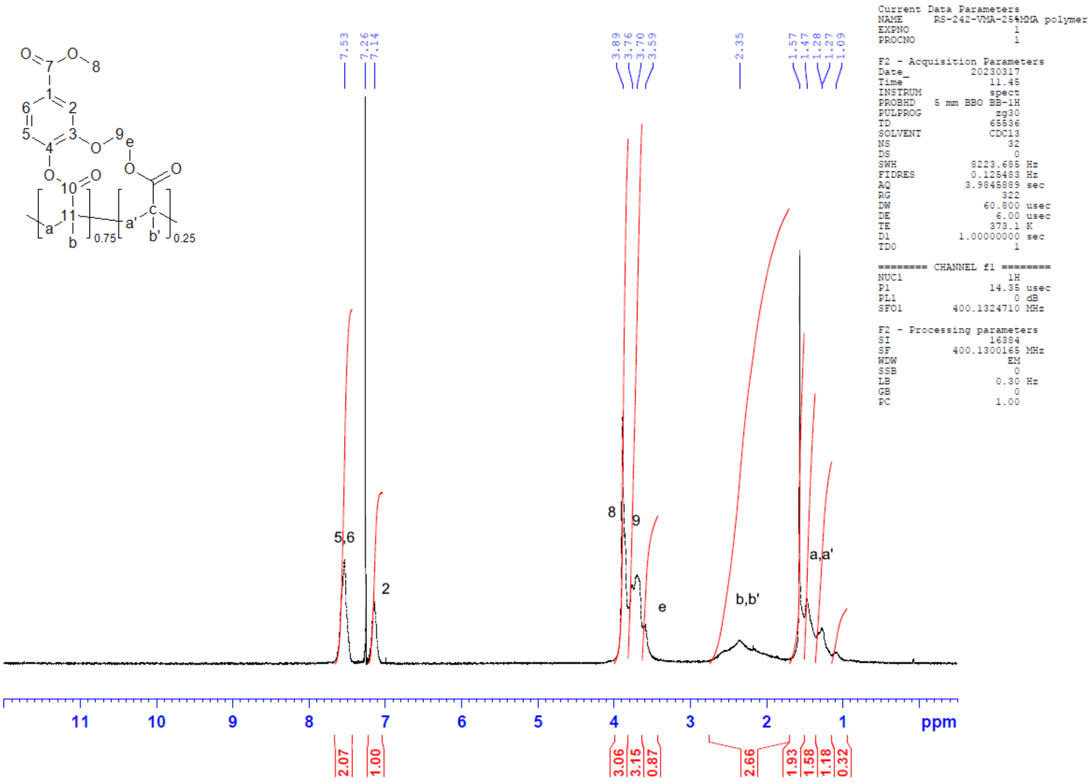

Figure S11. ^{1}H spectrum of PVMMA-25 measured in $CDCl_3$.

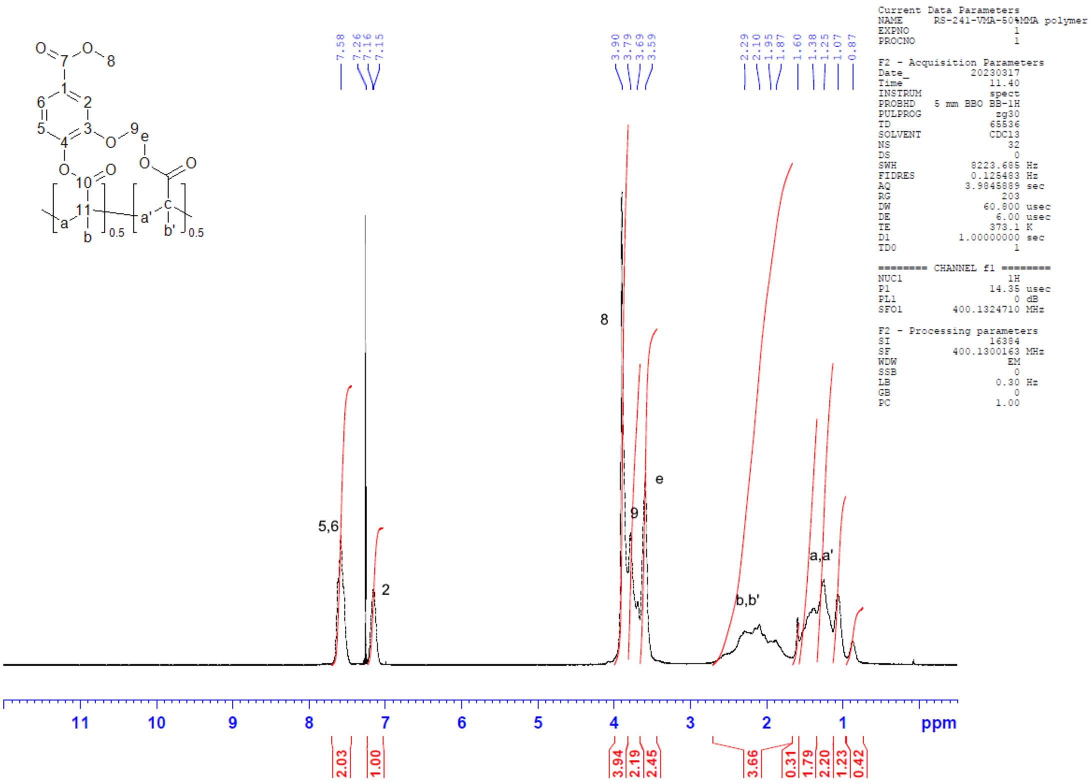

Figure S12. ^{1}H spectrum of PVMMA-50 measured in $CDCl_3$.

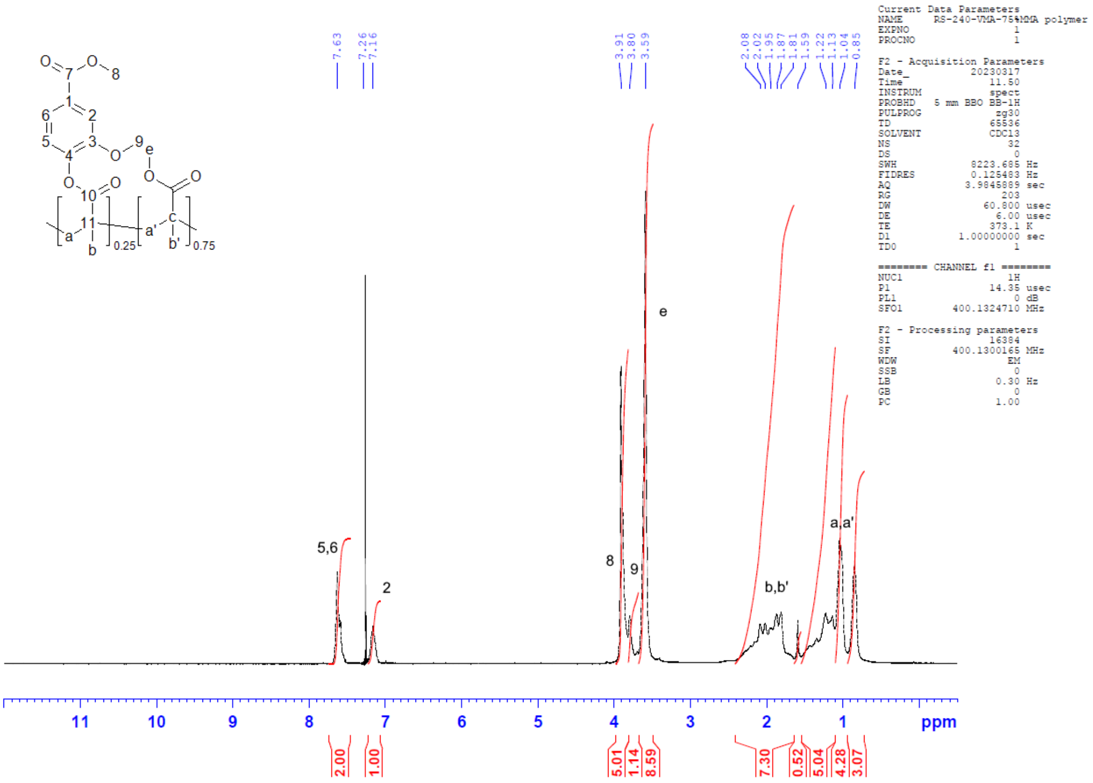

Figure S13. ^{1}H spectrum of PVMMA-75 measured in $CDCl_3$.

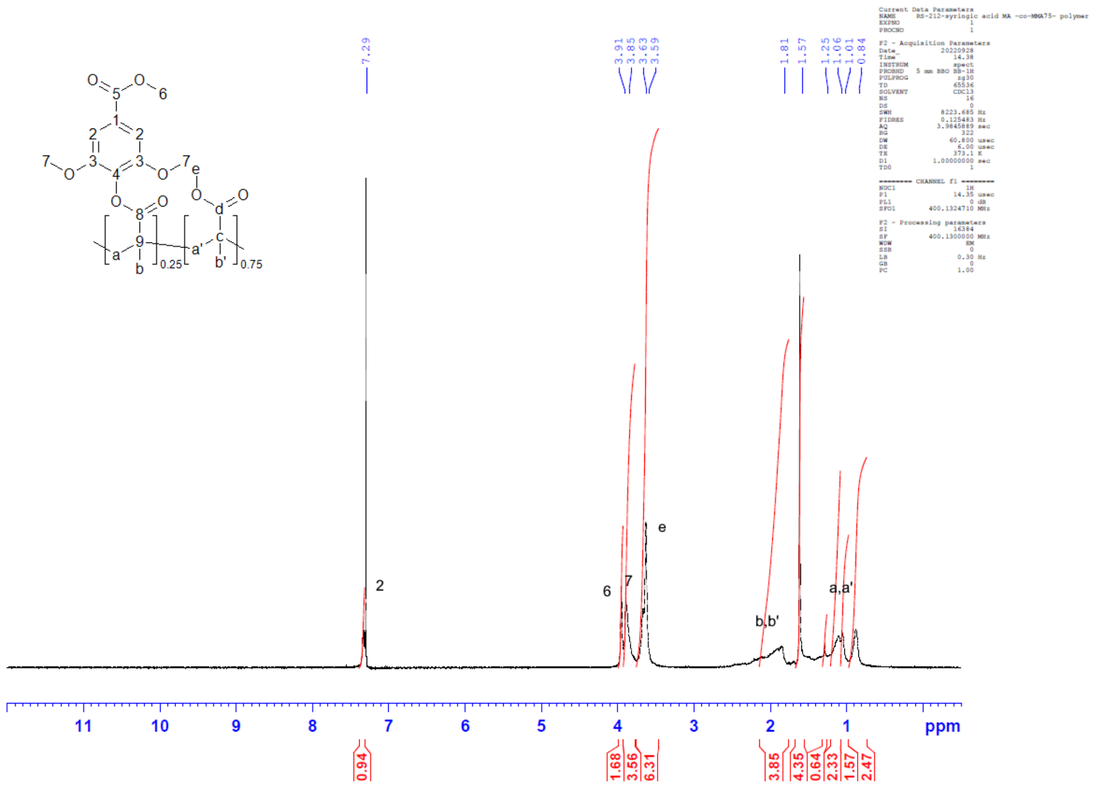

Figure S14. ^{1}H spectrum of PSMMA-75 measured in $CDCl_3$.

Polymer characterization

Thermal degradation

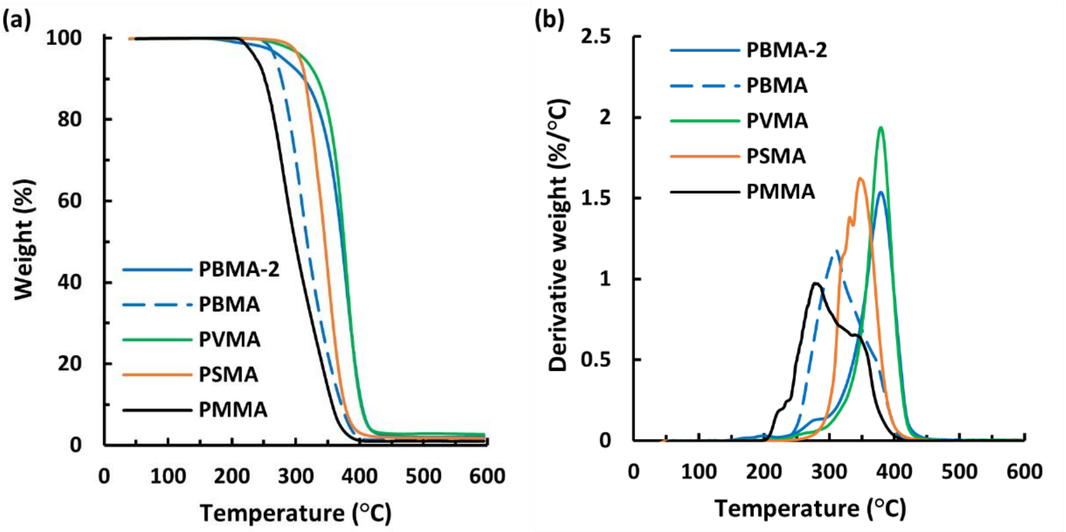

Figure S15. TGA (a) and corresponding differential TGA (b) traces of PBMA, PBMA-2, PVMA, PSMA, and PMMA.

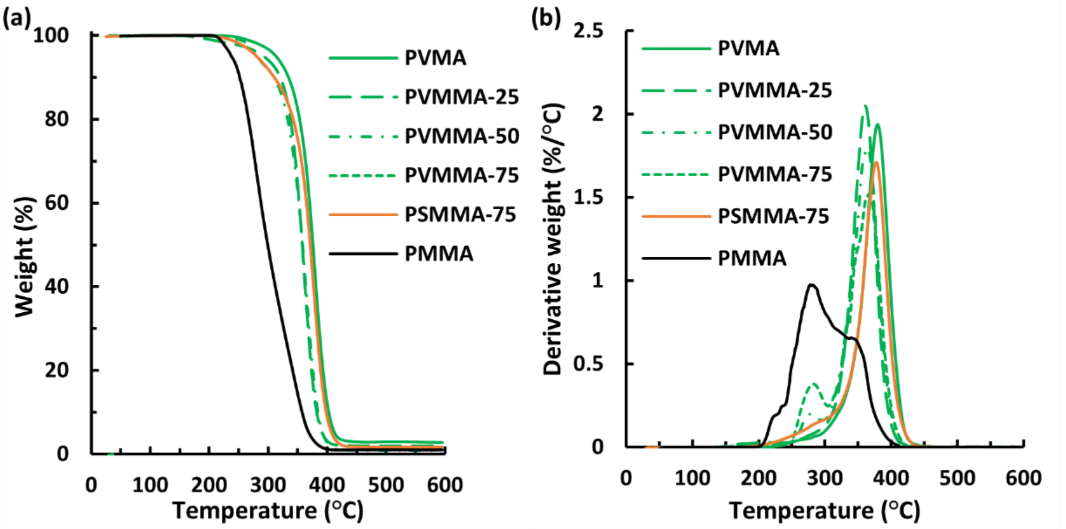

Figure S16. TGA (a) and differential TGA (b) traces of the homopolymers PVMA, PMMA, and the copolymers of VMA and SMA with MMA.

Rheology

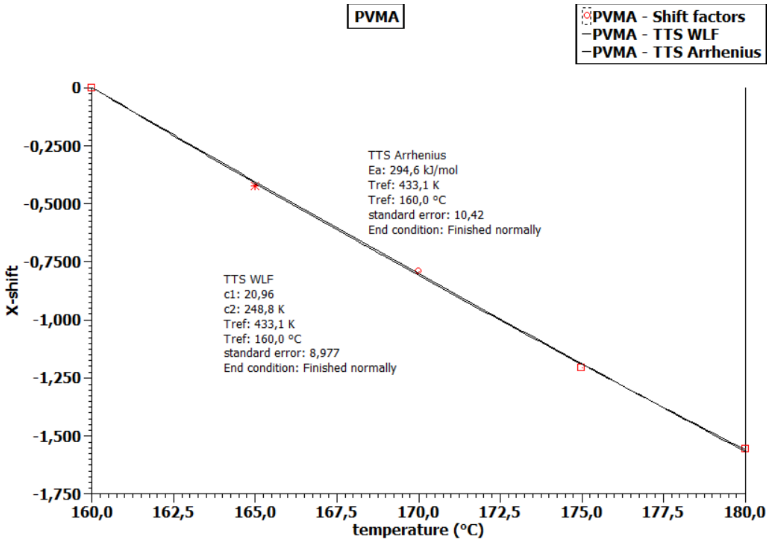

Figure S17. The plot of the shift factors obtained by time-temperature superposition of the frequency sweeps of PVMA relative to a reference temperature of 160 °C.

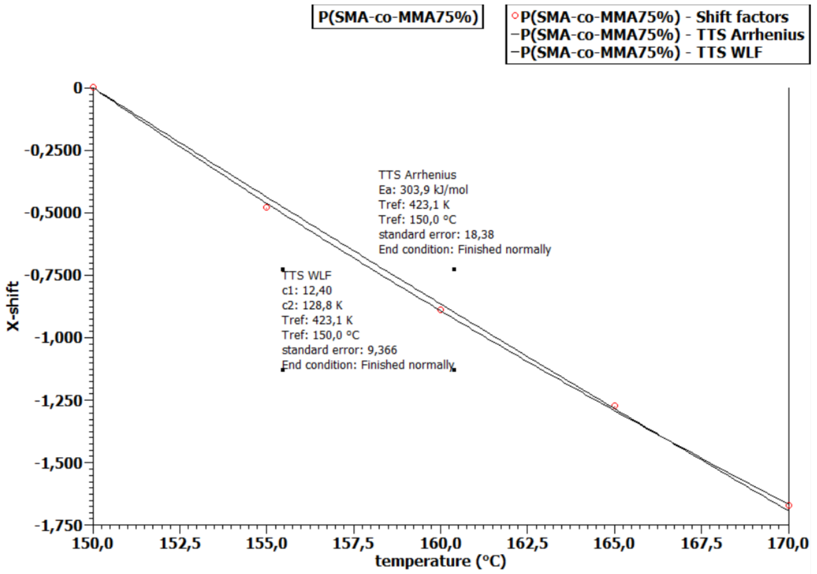

Figure S18. The plot of the shift factors obtained by time-temperature superposition of the frequency sweeps of PSMMA-75 relative to a reference temperature of 150 °C.

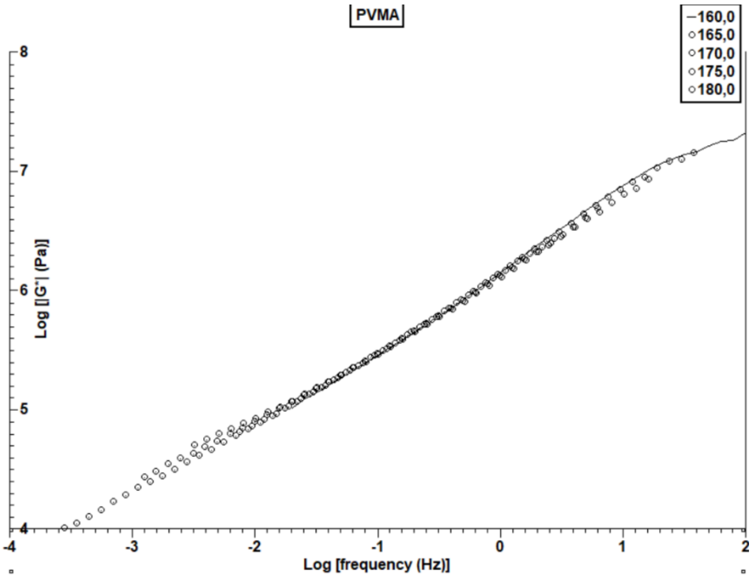

Figure S19. The complex dynamic shear modulus |G*| of PVMA plotted as a function of frequency and shifted by factor a_T to a reference temperature of 160 °C using time-temperature superposition.

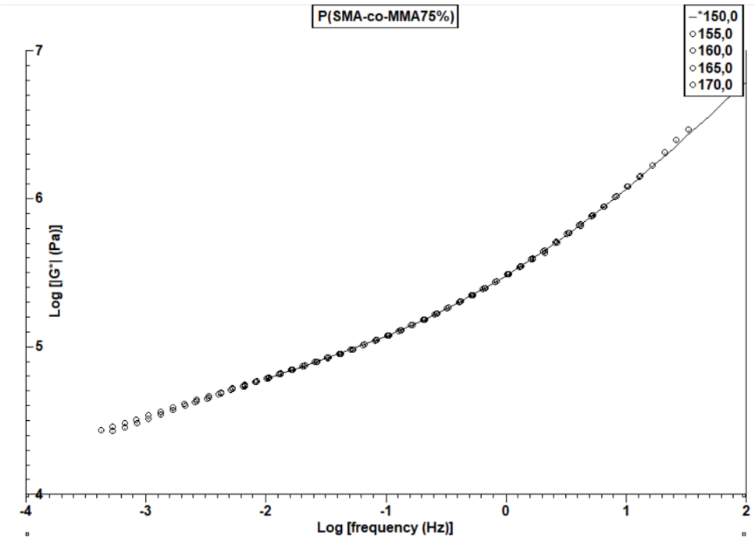

Figure S20. The complex dynamic shear modulus |G*| of PSMMA-75 was plotted as a function of frequency and shifted by a factor a_Z to a reference temperature of 150 °C using time-temperature superposition.

SEC curves

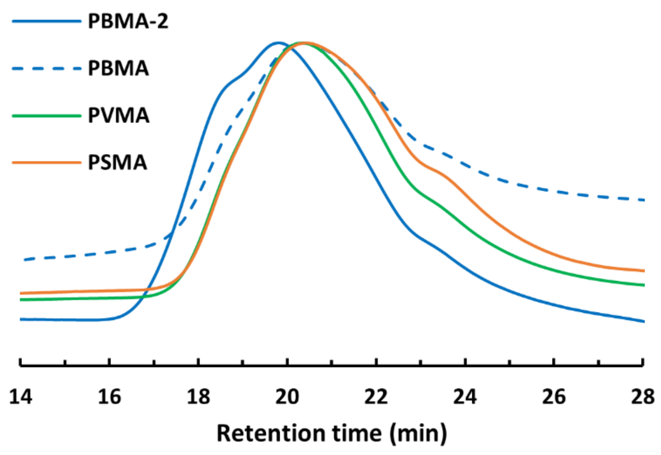

Figure S21. SEC curves of homopolymers measured in THF.

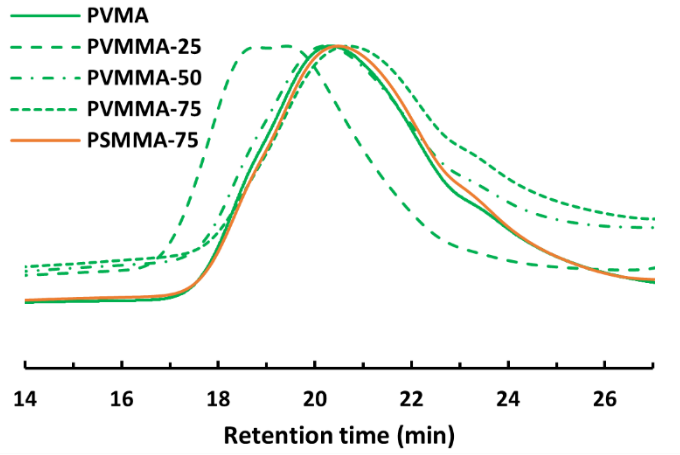

Figure S22. SEC curves of copolymers measured in THF.

Life cycle assessment and toxicity

General LCA framework

The combined framework integrating ex-ante and prospective LCA by Souza et al.[4] is applied to assess the environmental impacts of **VMA** and **SMA** produced via methacrylation using either methacrylic anhydride or methacryloyl chloride. A cradle-to-gate approach was considered, including the impacts of syringic acid and vanillin production from wood sugars to the monomer synthesis (Figure S23). The functional unit is 1 kg of monomer. The assessed environmental impacts are climate change, considering a 100-year global warming potential,[5] terrestrial acidification, and freshwater eutrophication using Recipe 2016 Midpoint (H)[6]. The

environmental impacts of the bio-based monomers are compared to fossil MMA and polystyrene.

This framework has three main phases. In Phase I: goal and scope definition, the functional unit and system boundaries are defined after the counterparts are identified. Finally, a value chain is designed. Phase II scales up the foreground data in six parts: a) process description, b) design of plant flow chart, c) identification of process synergies, d) improved yields, e) identification of side streams, and f) scaling up the operation units and energy requirements. In Phase III, the background data is projected a) identifying future narratives, b) selecting climate mitigation targets, c) transforming the background dataset, and d) connecting the projected background data with the scaled-up foreground data projecting background data.

All necessary equipment and machinery for industrial scale operations are identified during the process description and plant flow chart design (Figure S24). All the monomer production steps require heated and stirred reactors, besides the acylation in **VMA-acl** and **SMA-acl**, which require 2 h of cooling while adding methacryloyl chloride. The recovery of the primary solvents (e.g., methanol, ethyl acetate, and 2-MeTHF) and other chemicals used in the processes (e.g., sulfuric acid, triethyl amine) are identified as process synergies and can be reused. A 90% recovery rate is assumed when reusing triethyl amine, as some losses are expected when recovering the material from salt form. All other reused solvents and chemicals have a 97% recovery rate. Improved yields representative of industrial processes are considered for all four conversion pathways, assuming a 100% overall yield. The identification of side streams considers methacrylic acid as a co-product of **VMA-anh** and **SMA-anh** production, and the impacts are mass allocated among **VMA-anh** (66%) and methacrylic acid (34%), and **SMA-anh** (69%) and methacrylic acid (31%). No side streams are identified for **VMA-acl** and **SMA-acl** production. No fugitive emissions or wastes are identified for any of the four pathways. The energy required for heating, cooling, stirring, filtration, and recovery of materials is estimated based on Piccinno et al.[7], detailed in Equations S1 to S4 and Tables S3 to S6. A summary of all scaling-up procedures is presented in Table S7.

The foreground inventory was created considering the necessary inputs within the system boundaries. For vanillin, the input values reported by Borregaard,[8] which carry the environmental impacts of the biorefinery plant, have been used (Table S2). As there is currently no commercial production of syringic acid, the potential impacts from its production from Kraft lignin were therefore modeled following the reports by Khwanjaisakun[9] and Souza.[4] It is

assumed that the syringic acid production path could be similar to the vanillin/vanillic acid production path.

The electricity requirements come from the European average grid mix, and the required heat is from the combustion of wood residues. Background data comes from the Ecoinvent 3.8 database cut-off version.[10] To account for future socio-economic developments in the background supply chain, a projection of the background data is performed using PREMISE 1.2.5[11] with the narratives of the Shared Socioeconomic Pathway 2 – Middle of the road[12], under the climate policies of the Nationally Determined Contributions (NDC) for Europe. The complete life-cycle inventory is provided in Table S9.

The prospective LCA shows more limited contributions to reducing the impacts of the bio-based monomers. Compared to results based on ex-ante LCA, the average impacts for climate change, terrestrial acidification, and freshwater eutrophication decreased by 15, 12, and 24%, respectively. The background data used for this study accounts for our current fossil and linear chemical sector. A circular bioeconomy using renewable energy sources is key to achieving climate change mitigation and a sustainable future.[13,14] Further transformation of background data, including replacing fossil solvents and reactants (e.g., methacrylic anhydride, ethyl acetate, methanol) with cleaner pathways (e.g., bio-based solvents), could improve the results of the assessed bio-based monomers. For instance, some pathways for obtaining bio-based methacrylic acid have been recently investigated.[15,16] However, these cleaner pathways require higher maturity to be included in the database transformation.

Phase I: Goal and scope definition

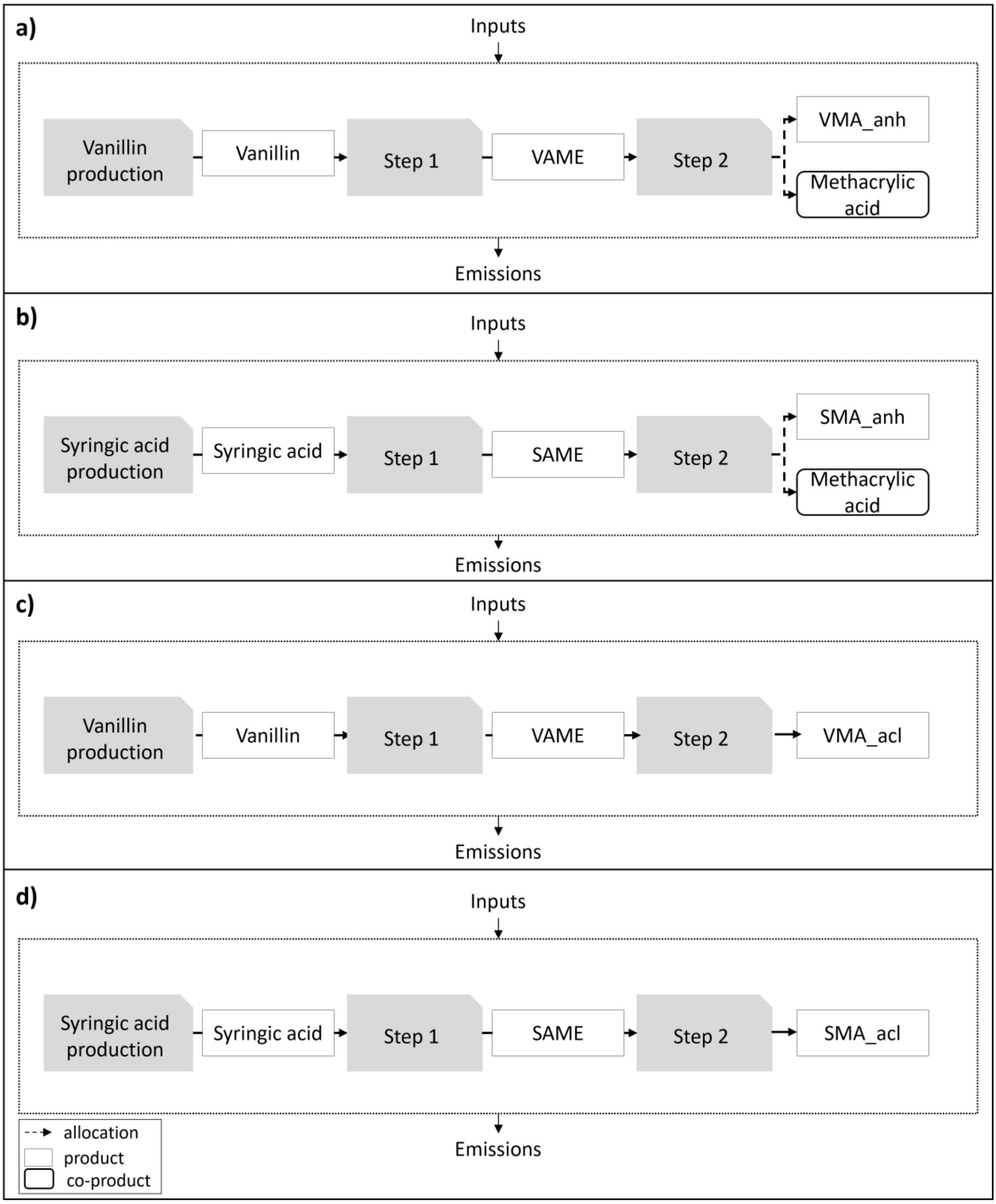

Figure S23: System boundaries and process description for VMA_anh (a), SMA_anh (b), VMA_acl (c), and SMA_acl (d).

*VAME (vanillic acid methyl ester), SAME (syringic acid methyl ester)

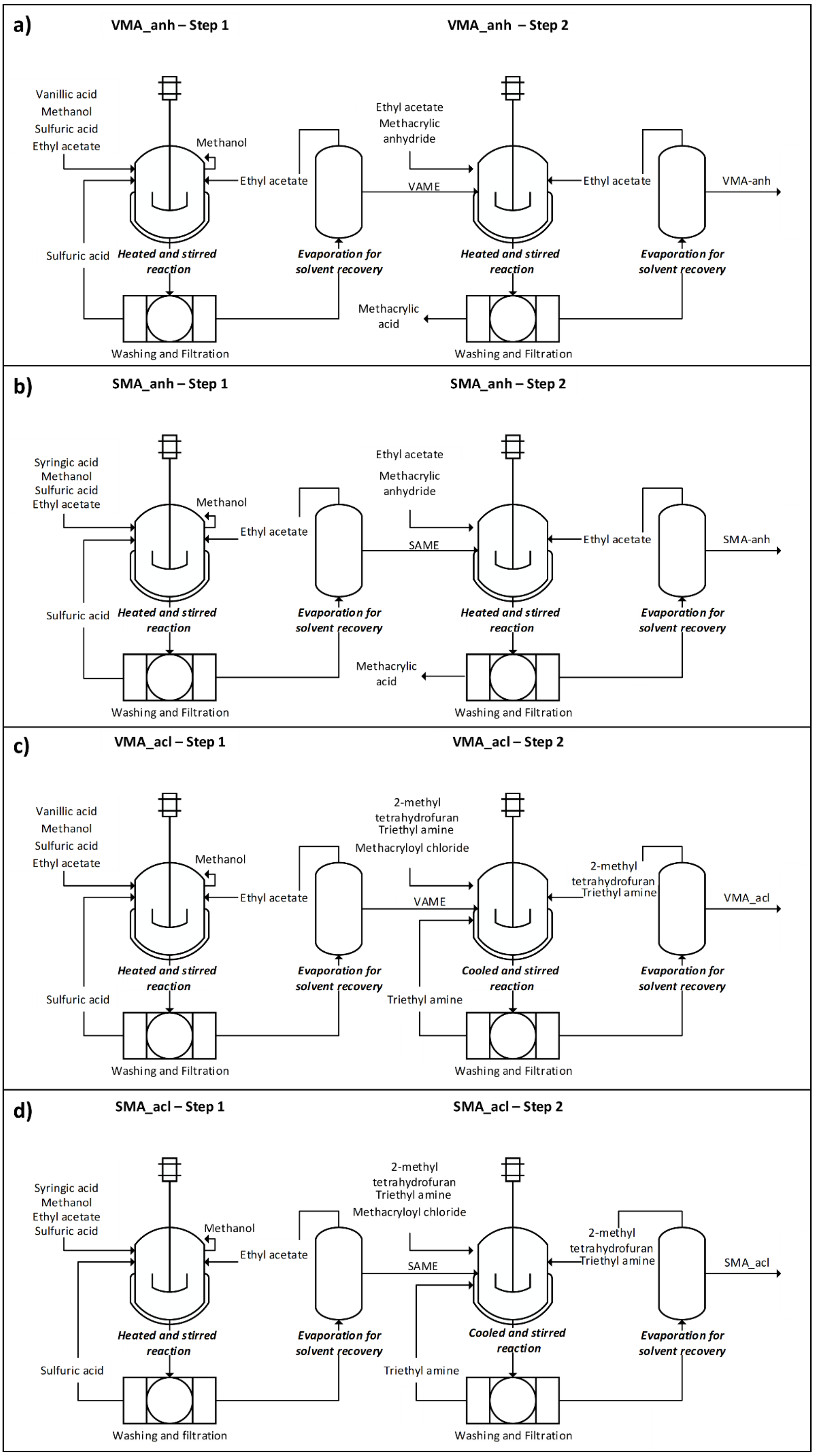

Figure S24: Process flow chart for production of VMA_anh (a), SMA_anh (b), VMA_acl (c), and SMA_acl (d).

Table S2: Environmental impacts of vanillin and syringic acid

Impact category	Vanillin[8]	Syringic acid[4]	Unit
Climate change	7.75E-01	7.89E+00	kg CO_2eq/kg (dry matter)
Terrestrial acidification	7.96E-03	4.22E-02	kg SO_2eq/kg (dry matter)
Freshwater eutrophication	2.96E-03	7.21E-03	kg Peq/kg (dry matter)

Source: Borregaard[8] and Souza et al.[4]

Phase II: Scaling up foreground data

$$Q_{react(10000\ l)} = \frac{C_p * m_{mix} * (T_r - 298.15\ K) + 15.333 * (T_r - 298.15\ K) * t}{0.75} \quad \text{Equation S1}$$

Where, Q_{react} is the heating energy required in joule (J), C_p specific heat capacity in joule per kg (J/kg·K), m_{mix} is the mass of the reaction mixture in kilogram (kg), T_r is the reaction temperature in kelvin (K) and t is the reaction time in seconds (s).

$$E_{stir(10000\ l)} = 0.083 * \rho_{mix} * t \quad \text{Equation S2}$$

Where, E_{stir} is the energy to stir the reactor in J, ρ_{mix} is the density of the mixture in kilogram per liter (kg/L), and t in is the reaction time, in seconds.

$$Q_{evap} = \frac{C_p * m_{mix} * (T_{boil} - T_0) + \Delta H_{vap} * m_{dist}}{0.97 * 0.9} \quad \text{Equation S3}$$

Where Q_{evap} is the evaporation energy required in J, C_p specific heat capacity in J/kg·K, m_{mix} the mass of the reaction mixture in kg, T_{boil} is the boiling temperature in K, T_0 is the initial temperature in K, ΔH_{vap} is the enthalpy of vaporization in kJ/kg and m_{dist} is the evaporated mass, in kg.

$$Q_{cool(10000\ l)} = \frac{C_p * m_{mix} * (T_r - T_{cool}) - 15.333 * (T_r - T_{cool}) * t}{0.75} \quad \text{Equation S4}$$

Where, Q_{cool} is the cooling energy required in joule (J), C_p specific heat capacity in joule per kg (J/kg·K), m_{mix} is the mass of the reaction mixture in kilogram (kg), T_r is the reaction temperature in kelvin (K), T_{cool} is the cooling temperature in kelvin (K), and t is the reaction time in seconds (s).

Table S3: Parameters to calculate heating energy requirements (Equation S1).

	Step	Main solvent	C_p (J/kg·K)	m_{mix} (kg)	T_r (K)	t (s)
VMA_anh	1	Methanol	2 481.3	7 784.3	353.2	57 600
	2	Ethyl acetate	1 929.4	8 161.5	323.2	57 600
SMA_anh	1	Methanol	2 481.3	7 784.3	353.2	57 600
	2	Ethyl acetate	1 929.4	8 161.5	323.2	57 600
VMA_acl	1	Methanol	2 481.3	8 154.9	353.2	57 600
	2	2-Me-THF**	1 855.9	7 937.5	353.2	57 600
SMA_acl	1	Methanol	2 481.3	7 784.3	353.2	57 600
	2	2-Me-THF**	1 855.9	8 011.2	353.2	57 600

*NIST Chemistry WebBook (https://webbook.nist.gov/chemistry/)
**2-methyltetrahydrofuran

Table S4: Parameters to calculate stirring energy requirements (Equation S2).

	Step	E_{stir} (kJ)	ρ_{mix} (kg/l)	t (s)
VMA_anh	1	4.2	0.9	57600
	2	4.4	0.9	57600
SMA_anh	1	4.2	0.9	57600
	2	4.4	0.9	57600
VMA_acl	1	4.4	0.9	57600
	2	4.2	0.9	57600
SMA_acl	1	4.2	0.9	57600
	2	4.3	0.9	57600

Table S5: Parameters to calculate evaporation energy requirements (Equation S3).

	Step	Recovered solvent	Q_{evap} (MJ)	C_p (J/kg·K)	m_{mix} (kg)	T_{boil} (K)*	T_0 (K)	ΔH_{vap} (J/kg)*	m_{dist} (kg)
VMA_anh	1	Ethyl acetate	1 053	1 929	7 784	350	353	362 501	7 784
	2	Ethyl acetate	3 674	1 929	7 973	350	323	362 501	7 973
SMA_anh	1	Ethyl acetate	1 053	1 929	7 784	350	353	362 501	7 784
	2	Ethyl acetate	3 674	1 929	7 973	350	323	362 501	7 973
VMA_acl	1	Ethyl acetate	1 209	1 929	8 155	350	353	362 501	8 155
	2	2-Me-THF**	3 291	1 856	7 938	352	353	394 742	7 938
SMA_acl	1	Ethyl acetate	1 053	1 929	7 784	350	353	362 501	7 784
	2	2-Me-THF**	3 360	1 856	8 011	352	353	394 742	8 011

*NIST Chemistry WebBook (https://webbook.nist.gov/chemistry/)
**2-methyltetrahydrofuran

Table S6: Parameters to calculate cooling energy requirements (Equation S4)

	Step	Main solvent	Q_{cool} (MJ)	C_p (J/kg·K)*	m_{mix} (kg)	T_r (K)	T_{cool} (K)	t (s)
VMA_acl	2	2-Me-THF**	1481	1856	7938	353	273	7200
SMA_acl	2	2-Me-THF**	1494	1856	8011	353	273	7200

*NIST Chemistry WebBook (https://webbook.nist.gov/chemistry/)
**2-methyltetrahydrofuran

Table S7: Procedures applied to scale up laboratory data to an industrial scale (ex-ante LCA) to produce bio-based VMA.

	Description	VMA_anh	SMA_anh	VMA_acl	SMA_acl
**Part I: Process description**	Flowchart of the process	Figure S21			
**Part II: Plant flow chart**	Identification of all equipment and machinery	Figure S22			
**Part III: Process synergies**	90-97% recovery of solvents and chemicals	Methanol, Ethyl acetate, sulfuric acid	Methanol, Ethyl acetate, sulfuric acid	Methanol, Ethyl acetate, sulfuric acid, 2-MeTHF, triethyl amine	Methanol, Ethyl acetate, sulfuric acid, 2-MeTHF, triethyl amine
	Removal of lab-scale procedures	Washing with aq. solutions.	Washing with aq. solutions.	Washing with aq. solutions.	Washing with aq. solutions.
**Part IV: Improved yields**	100% conversion of reactants into targeted products, no waste				
**Part V: Side streams**	Identification of co-products	Methacrylic acid	Methacrylic acid	None	None
**Part VI: Scaling-up[1]**	· Filtration/centrifuge	5.5 kWh/t filtrate			
	· Pumping	55 J/kg of pumped material			
	· Reaction heating	Equation S1			
	· Stirring	Equation S2			
	· Solvent and chemical recovery: evaporation and filtration	Equation S3 Targets: Ethyl acetate. Methanol evaporates during the heated reaction	Equation S3 Targets: Ethyl acetate. Methanol evaporates during the heated reaction	Equation S3 Targets: Ethyl acetate and 2-MeTHF. Methanol evaporates during the heated reaction	Equation S3 Targets: Ethyl acetate and 2-MeTHF. Methanol evaporates during the heated reaction
	Reaction cooling	None	None	Equation S4	

[1]based on Piccino et al.[7]

Sensitivity analysis

A sensitivity analysis was performed to account for the most uncertain parameters during the scaling-up of foreground data (Phase II). Three main parameters were selected: overall yield, recovery of solvents and the amount of solvent used in reactions (Table S8). During the scaling-up, Phase II - Part IV, we assume an optimistic yield improvement; for the sensitivity analysis, the practical yields of each step are considered for all the monomers in a low yield scenario. The second parameter is the recovery rate of solvents, a conservative approach is taken, considering a recovery rate 15% lower than the default values used in Phase II - Part III. Higher rates of recovery were considered for methanol, ethyl acetate, petrol ether, reaching up to

99.5%, and for 2-methyl-tetrahydrofuran and triethylamine, reaching 97%. Finally, we assess the environmental impacts when using 30% less solvent input compared to the default values.

Table S8. Parameters for sensitivity analysis of bio-based monomers VMA-anh, VMA-acl, SMA-anh and SMA-acl

**Monomer**	**Step**	**Yield scenarios**		**Recovery rate scenarios of solvents and reagents**				**Amount of solvent used**	
		**Low yield**	**Default***	**Solvents and chemicals**	**Lower recovery rate**	**Default***	**Higher recovery rate**	**Lower solvent use****	**Default***
VMA-anh	1	91.6%	100%	Methanol	82.5%	97.0%	99.5%	70%	100%
				Ethyl acetate	82.5%	97.0%	99.5%	N/A	N/A
				Sulfuric acid	82.5%	97.0%	97.0%	N/A	N/A
	2	50.0%	100%	Ethyl acetate	82.5%	97.0%	99.5%	70%	100%
				Petrol ether	82.5%	97.0%	99.5%	N/A	N/A
SMA-anh	1	97.0%	100%	Methanol	82.5%	97.0%	99.5%	70%	100%
				Ethyl acetate	82.5%	97.0%	99.5%	N/A	N/A
				Sulfuric acid	82.5%	97.0%	97.0%	N/A	N/A
	2	64.6%	100%	Ethyl acetate	82.5%	97.0%	99.5%	70%	100%
				Petrol ether	82.5%	97.0%	99.5%	N/A	N/A
VMA-acl	1	91.6%	100%	Methanol	82.5%	97.0%	99.5%	70%	100%
				Ethyl acetate	82.5%	97.0%	99.5%	N/A	N/A
				Sulfuric acid	82.5%	97.0%	97.0%	N/A	N/A
	2	90.0%	100%	Triethyl amine	76.5%	90.0%	97.0%	N/A	N/A
				2-Me-THF	76.5%	90.0%	97.0%	70%	100%
SMA-acl	1	97.0%	100%	Methanol	82.5%	97.0%	99.5%	70%	100%
				Ethyl acetate	82.5%	97.0%	99.5%	N/A	N/A
				Sulfuric acid	82.5%	97.0%	97.0%	N/A	N/A
	2	92.4%	100%	Triethyl amine	76.5%	90.0%	97.0%	N/A	N/A
				2-Me-THF	76.5%	90.0%	97.0%	70%	100%

*The values presented on Figure 4 and Figure S25; N/A: not applicable
**This means 30% less solvent was used in this scenario compared to the default value.

Phase III: Projection of background data

PREMISE 1.2.5 transforms the Ecoinvent 3.8 database[10] into future background systems provided by the Integrated Assessment Model REMIND[17]. The default transformations of PREMISE are power generation, cement production, steel production, transport, and fuels.[11]

The addition of new technology and efficiency enhancements (such as biofuels, carbon capture and storage, cleaner energy sources, etc.) are the main changes.

The Shared Socioeconomic Pathway – Middle of the road (SSP2)[12] is the selected future narrative and it extrapolates current socioeconomic trends and incorporate historical patterns of the economy.[12] We selected the Nationally Determined Contribution (NDC) for Europe as climate mitigation target, coupled with SSP2.

Table S9: Life-cycle inventory to produce VMA_anh and VMA_acl from vanillin and SMA_anh and SMA_acl from syringic acid.

**Inputs**	**Lab-scale**	**Ex-ante LCA**					**Prospective LCA**			**Unit**
		**Process synergies I**	**Process synergies II**	**Improved yields**	**Side-streams**	**Scaling up**	**2030**	**2040**	**2050**	
**VMA_anh - Step 1**										
Vanillin	1.01	1.01	1.01	0.92	0.92	0.92	0.92	0.92	0.92	kg/kg VAME_1
Methanol	7.20	0.22	0.22	0.22	0.22	0.22	0.22	0.22	0.22	kg/kg VAME_1
Sulfuric acid	0.33	0.33	0.01	0.01	0.01	0.01	0.01	0.01	0.01	kg/kg VAME_1
Sodium bicarbonate	0.0036	0.00	-	-	-	-	-	-	-	kg/kg VAME_1
Ethyl acetate	4.10	4.10	0.12	0.12	0.12	0.12	0.12	0.12	0.12	kg/kg VAME_1
Magnesium	0.91	0.91	0.91	0.91	0.91	0.91	0.91	0.91	0.91	kg/kg VAME_1
Heat*	-	-	-	-	-	4.27	4.27	4.27	4.27	MJ/kg VAME_1
Electricity, high voltage	-	-	-	-	-	0.07	0.07	0.07	0.07	kWh/kg VAME_1
**VMA_anh - Step 2**										
VAME	1.46	1.46	1.46	0.73	0.48	0.48	0.48	0.48	0.48	kg/kg VMA_anh
Ethyl acetate	45.97	1.38	1.38	1.38	0.90	0.90	0.90	0.90	0.90	kg/kg VMA_anh
Magnesium sulfate	1.46	1.46	1.46	1.46	0.96	0.96	0.96	0.96	0.96	kg/kg VMA_anh
Ethyl acetate	32.83	0.98	0.98	0.98	0.65	0.65	0.65	0.65	0.65	kg/kg VMA_anh
Methacrylic anhydride**	1.97	1.97	1.97	0.64	0.42	0.42	0.42	0.42	0.42	kg/kg VMA_anh
Sodium bicarbonate	0.010	0.01	-	-	-	-	-	-	-	kg/kg VMA_anh
Sodium chloride,	0.24	0.24	-	-	-	-	-	-	-	kg/kg VMA_anh
Chemical, inorganic	0.02	0.02	-	-	-	-	-	-	-	kg/kg VMA_anh
Chemical, inorganic	23.77	23.77	0.71	0.71	0.47	0.47	0.47	0.47	0.47	kg/kg VMA_anh
Electricity, high voltage	-	-	-	-	-	0.16	0.16	0.16	0.16	kg/kg VMA_anh
Heat*	-	-	-	-	-	14.95	14.95	14.95	14.95	kg/kg VMA_anh
**VMA_acl - Step 1**										
Vanillic acid	1.01	1.01	1.01	0.92	0.92	0.92	0.92	0.92	0.92	kg/kg VAME_2

Methanol	7.20	0.22	0.22	0.22	0.22	0.22	0.22	0.22	0.22	kg/kg VAME_2
Sulfuric acid	0.33	0.33	0.01	0.01	0.01	0.01	0.01	0.01	0.01	kg/kg VAME_2
Sodium bicarbonate	0.0023	0.00	-	-	-	-	-	-	-	ml/kg VAME_2
Ethyl acetate	4.10	4.10	0.12	0.12	0.12	0.12	0.12	0.12	0.12	kg/kg VAME_2
Magnesium sulfate	0.91	0.91	0.91	0.91	0.91	0.91	0.91	0.91	0.91	kg/kg VAME_2
Heat	-	-	-	-	-	4.27	4.27	4.27	4.27	MJ/kg VAME_2
Electricity	-	-	-	-	-	0.07	0.07	0.07	0.07	kWh/kg VAME_2
**VMA_acl - Step 2**										
VAME	0.82	0.82	0.82	0.73	0.73	0.73	0.73	0.73	0.73	kg/kg VMA_acl
Methacryloyl chloride	0.49	0.49	0.49	0.44	0.44	0.44	0.44	0.44	0.44	kg/kg VMA_acl
Triethyl amine	0.54	0.54	0.05	0.05	0.05	0.05	0.05	0.05	0.05	kg/kg VMA_acl
2-MeTHF	13.61	1.36	1.36	1.36	1.36	1.36	1.36	1.36	1.36	kg/kg VMA_acl
Sodium hydroxide	0.19	0.19	0.19	0.19	0.19	0.19	0.19	0.19	0.19	kg/kg VMA_acl
Sodium bicarbonate	0.04	0.04	-	-	-	-	-	-	-	L/kg VMA_acl
Brine	0.22	0.22	-	-	-	-	-	-	-	L/kg VMA_acl
Magnesium sulfate	0.40	0.40	0.40	0.40	0.40	0.40	0.40	0.40	0.40	kg/kg VMA_acl
Heat	-	-	-	-	-	6.98	6.98	6.98	6.98	MJ/kg VMA_acl
Electricity	-	-	-	-	-	0.07	0.07	0.07	0.07	kWh/kg VMA_acl
Cooling	-	-	-	-	-	2.36	2.36	2.36	2.36	MJ/kg VMA_acl
**SMA_anh - Step 1**										
Syringic acid	0.95	0.95	0.95	0.93	0.93	0.93	0.93	0.93	0.93	kg/kg SAME_1
Methanol	7.21	0.22	0.22	0.22	0.22	0.22	0.22	0.22	0.22	kg/kg SAME_1
Sulfuric acid	0.33	0.33	0.01	0.01	0.01	0.01	0.01	0.01	0.01	kg/kg SAME_1
Sodium bicarbonate	0.0036	0.00	-	-	-	-	-	-	-	L/kg SAME_1
Ethyl acetate	4.11	4.11	0.12	0.12	0.12	0.12	0.12	0.12	0.12	kg/kg SAME_1
Magnesium sulfate	0.91	0.91	0.91	0.91	0.91	0.91	0.91	0.91	0.91	kg/kg SAME_1
Heat	-	-	-	-	-	3.90	3.90	3.90	3.90	MJ/kg SAME_1
Electricity	-	-	-	-	-	0.07	0.07	0.07	0.07	kWh/kg SAME_1
**SMA_anh - Step 2**										
SAME	1.17	1.17	1.17	0.76	0.52	0.52	0.52	0.52	0.52	kg/kg SMA_anh
Methacrylic anhydride	0.87	0.87	0.87	0.55	0.38	0.38	0.38	0.38	0.38	kg/kg SMA_anh
Dimethyl-aminopyridin	21.44	21.44	0.64	-	-	-	-	-	-	kg/kg SMA_anh

Ethyl acetate	69.07	2.07	2.07	2.07	1.44	1.44	1.44	1.44	1.44	kg/kg SMA_anh
Sodium bicarbonate	0.03	0.03	-	-	-	-	-	-	-	l/kg SMA_anh
Brine	0.42	0.42	-	-	-	-	-	-	-	l/kg SMA_anh
Ethyl acetate	17.27	0.52	0.52	0.52	0.36	0.36	0.36	0.36	0.36	kg/kg SMA_anh
Petrol ether	12.50	12.50	0.38	0.38	0.26	0.26	0.26	0.26	0.26	kg/kg SMA_anh
Magnesium sulfate	1.53	1.53	1.53	1.53	1.06	1.06	1.06	1.06	1.06	kg/kg SMA_anh
Heat	-	-	-	-	-	14.47	14.47	14.47	14.47	MJ/kg SMA_anh
Electricity	-	-	-	-	-	0.07	0.07	0.07	0.07	kWh/kg SMA_anh
**SMA_acl - Step 1**										
Syringic acid	0.95	0.95	0.95	0.93	0.93	0.93	0.93	0.93	0.93	kg/kg SAME_2
Methanol	7.21	0.22	0.22	0.22	0.22	0.22	0.22	0.22	0.22	kg/kg SAME_2
Sulfuric acid	0.33	0.33	0.01	0.01	0.01	0.01	0.01	0.01	0.01	kg/kg SAME_2
Sodium bicarbonate	0.00	0.00	-	-	-	-	-	-	-	l/kg SAME_2
Ethyl acetate	4.11	4.11	0.12	0.12	0.12	0.12	0.12	0.12	0.12	kg/kg SAME_2
Magnesium sulfate	0.91	0.91	0.91	0.91	0.91	0.91	0.91	0.91	0.91	kg/kg SAME_2
Heat	-	-	-	-	-	3.90	3.90	3.90	3.90	MJ/ kg SAME_2
Electricity	-	-	-	-	-	0.07	0.07	0.07	0.07	kWh/ kg SAME_2
**SMA_acl - Step 2**										
SAME	0.82	0.82	0.82	0.76	0.76	0.76	0.76	0.76	0.76	kg/kg SMA_acl
Methacryloyl chloride	0.43	0.43	0.43	0.39	0.39	0.39	0.39	0.39	0.39	kg/kg SMA_acl
Triethyl amine	0.43	0.43	0.04	0.04	0.04	0.04	0.04	0.04	0.04	kg/kg SMA_acl
2-MeTHF	15.11	1.51	1.51	1.51	1.51	1.51	1.51	1.51	1.51	kg/kg SMA_acl
Sodium hydroxide	0.16	0.16	0.16	0.16	0.16	0.16	0.16	0.16	0.16	kg/kg SMA_acl
Sodium bicarbonate	0.04	0.04	-	-	-	-	-	-	-	l/kg SMA_acl
Brine	0.25	0.25	-	-	-	-	-	-	-	L/kg SMA_acl
Magnesium sulfate	0.44	0.44	0.44	0.44	0.44	0.44	0.44	0.44	0.44	kg/kg SMA_acl
Heat	-	-	-	-	-	7.06	7.06	7.06	7.06	MJ/kg SMA_acl
Electricity	-	-	-	-	-	0.07	0.07	0.07	0.07	kWh/kg SMA_acl
Cooling	-	-	-	-	-	2.37	2.37	2.37	2.37	MJ/kg SMA_acl

*Climate change impacts of heat from wood residues is 16.0 g CO2eq./MJ heat under ex-ante LCA; for prospective LCA, the impacts are 9.3 gCO2eq/MJ in 2030, 7.8 g CO2eq./MJ in 2040 and 6.8 g CO2eq./MJ for 2050.

**Climate change impacts of methacrylic acid are used by means of simplification as no data for methacrylic anhydride is available.

Results

Table S10: Environmental impacts of VMA and fossil counterparts.

<table>
<tr><th colspan="2">Impact categories</th><th>Climate change (kg CO_2 eq/kg monomer)</th><th>Terrestrial acidification (kg SO_2 eq/t monomer)</th><th>Freshwater eutrophication (kg P eq/t monomer)</th></tr>
<tr><td colspan="5">VMA_anh</td></tr>
<tr><td colspan="2">Lab-Scale</td><td>280.46</td><td>1 188.65</td><td>91.32</td></tr>
<tr><td rowspan="5">Ex-ante LCA</td><td>Process synergies I</td><td>79.30</td><td>443.14</td><td>28.08</td></tr>
<tr><td>Process synergies II</td><td>21.76</td><td>105.73</td><td>7.23</td></tr>
<tr><td>Improved yields</td><td>12.53</td><td>58.38</td><td>4.45</td></tr>
<tr><td>Side streams</td><td>8.24</td><td>38.38</td><td>2.93</td></tr>
<tr><td>Scaling-up</td><td>8.58</td><td>40.97</td><td>3.18</td></tr>
<tr><td rowspan="3">Prospective LCA</td><td>2030</td><td>6.94</td><td>33.49</td><td>1.89</td></tr>
<tr><td>2040</td><td>6.54</td><td>32.46</td><td>1.74</td></tr>
<tr><td>2050</td><td>6.34</td><td>31.93</td><td>1.67</td></tr>
<tr><td colspan="5">VMA_acl</td></tr>
<tr><td colspan="2">Lab-Scale</td><td>95.04</td><td>484.53</td><td>37.25</td></tr>
<tr><td rowspan="5">Ex-ante LCA</td><td>Process synergies I</td><td>22.64</td><td>106.90</td><td>8.63</td></tr>
<tr><td>Process synergies II</td><td>12.32</td><td>65.98</td><td>5.12</td></tr>
<tr><td>Improved yields</td><td>11.84</td><td>63.04</td><td>4.88</td></tr>
<tr><td>Side streams</td><td>11.84</td><td>63.04</td><td>4.88</td></tr>
<tr><td>Scaling-up</td><td>12.17</td><td>64.79</td><td>5.04</td></tr>
<tr><td rowspan="3">Prospective LCA</td><td>2030</td><td>11.36</td><td>61.17</td><td>4.28</td></tr>
<tr><td>2040</td><td>11.21</td><td>60.76</td><td>4.24</td></tr>
<tr><td>2050</td><td>11.14</td><td>60.55</td><td>4.21</td></tr>
<tr><td colspan="5">SMA_anh</td></tr>
<tr><td colspan="2">Lab-Scale</td><td>315.08</td><td>1 370.41</td><td>108.58</td></tr>
<tr><td rowspan="5">Ex-ante LCA</td><td>Process synergies I</td><td>96.53</td><td>558.87</td><td>39.47</td></tr>
<tr><td>Process synergies II</td><td>23.63</td><td>113.76</td><td>12.67</td></tr>
<tr><td>Improved yields</td><td>17.05</td><td>78.59</td><td>8.76</td></tr>
<tr><td>Side streams</td><td>11.76</td><td>54.16</td><td>6.02</td></tr>
<tr><td>Scaling-up</td><td>12.07</td><td>56.54</td><td>6.23</td></tr>
<tr><td rowspan="3">Prospective LCA</td><td>2030</td><td>10.44</td><td>49.15</td><td>4.97</td></tr>
<tr><td>2040</td><td>10.05</td><td>48.12</td><td>4.82</td></tr>
<tr><td>2050</td><td>9.85</td><td>47.59</td><td>4.75</td></tr>
<tr><td colspan="5">SMA_acl</td></tr>
<tr><td colspan="2">Lab-Scale</td><td>108.65</td><td>557.45</td><td>45.49</td></tr>
<tr><td>Ex-ante LCA</td><td>Process synergies I</td><td>28.65</td><td>139.30</td><td>13.75</td></tr>
</table>

	Process synergies II	18.23	94.73	10.17
	Improved yields	17.38	90.37	9.58
	Side streams	17.38	90.37	9.58
	Scaling-up	17.71	92.11	9.75
Prospective LCA	2030	16.95	88.70	9.02
	2040	16.81	88.33	8.98
	2050	16.75	88.13	8.96
Fossil	Styrene	2.94	9.58	0.52
	MMA	6.93	30.47	0.30

Table S11: Impacts of bio-based 2-MeTHF, fossil tetrahydrofuran and fossil ethyl acetate

**Impacts**	Bio-based 2-MeTHF (corn stover)[18]	Tetrahydrofuran[10]	Ethyl acetate[10]
Climate Change (kg CO2 eq/kg product)	5.62	5.95	2.82
Terrestrial Acidification (kg SO_2 eq/t product)	30.00	24.43	12.34
Freshwater Eutrophication (kg P eq/t product)	2.31	2.04	1.02

Table S12. Sensitivity analysis of the environmental impacts of VMA-anh, VMA-acl, SMA-anh and SMA-acl

**Climate change impacts (kg CO_2 eq/kg monomer)**					
**Monomers**	**Low recovery rate**	**High recovery rate**	**Low solvent use**	**Low yield**	**Default***
VMA_anh	47.77	4.80	6.30	10.11	8.58
SMA_anh	50.80	8.04	9.31	17.49	12.07
VMA_acl	24.41	6.45	6.61	12.36	12.17
SMA_acl	31.07	11.36	11.53	18.33	17.71
**Terrestrial acidification (kg SO_2 eq/t monomer)**					
**Monomers**	**Low recovery rate**	**High recovery rate**	**Low solvent use**	**Low yield**	**Default***
VMA_anh	206.27	25.09	32.47	52.34	40.97
SMA_anh	211.01	40.52	46.28	85.09	56.54
VMA_acl	126.94	34.92	35.53	66.32	64.79
SMA_acl	160.23	58.90	59.51	95.34	92.11
**Freshwater eutrophication (kg P eq/t monomer)**					
**Monomers**	**Low recovery rate**	**High recovery rate**	**Low solvent use**	**Low yield**	**Default***
VMA_anh	16.08	1.94	2.45	4.55	3.18
SMA_anh	18.81	4.93	5.36	11.00	6.23
VMA_acl	9.82	2.73	2.79	5.23	5.04
SMA_acl	14.97	7.18	7.24	10.29	9.75

*The values presented on Figure S25

Visualization of results

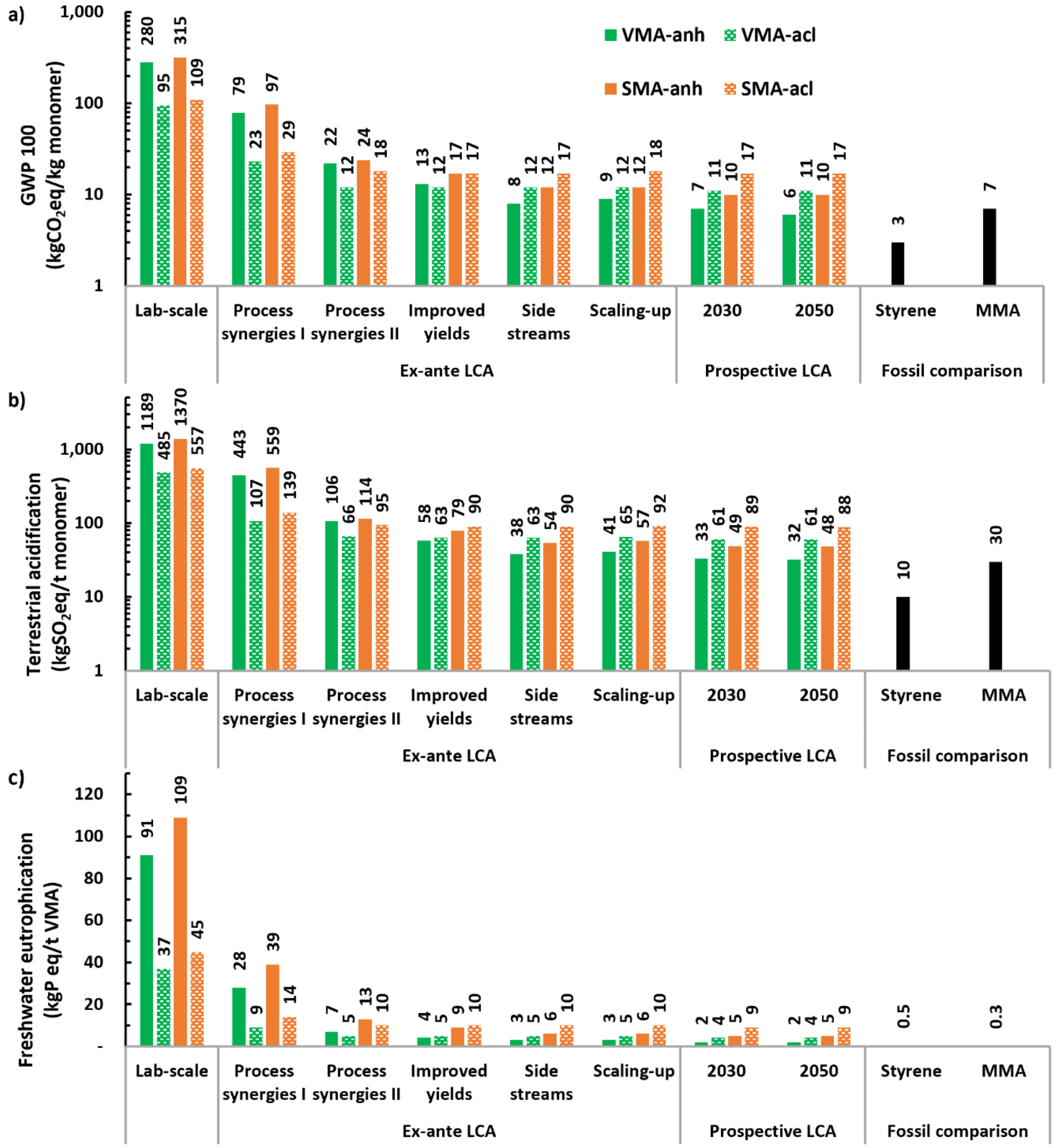

Figure S25. The environmental impacts of VMA and SMA using two different methods (VMA-anh, VMA-acl, SMA-anh, and SMA-acl) and fossil counterparts. Climate change (a), terrestrial acidification (b), and freshwater eutrophication (c). Process synergies I: Recovery of the primary solvent; Process synergies II: Recovery of other chemicals; Improved yields: No reactant wastes, at maximum product yield. Side streams: Identified co-products with the allocation of impacts. Scaling-up: Required energy and its indirect emissions of monomer production.

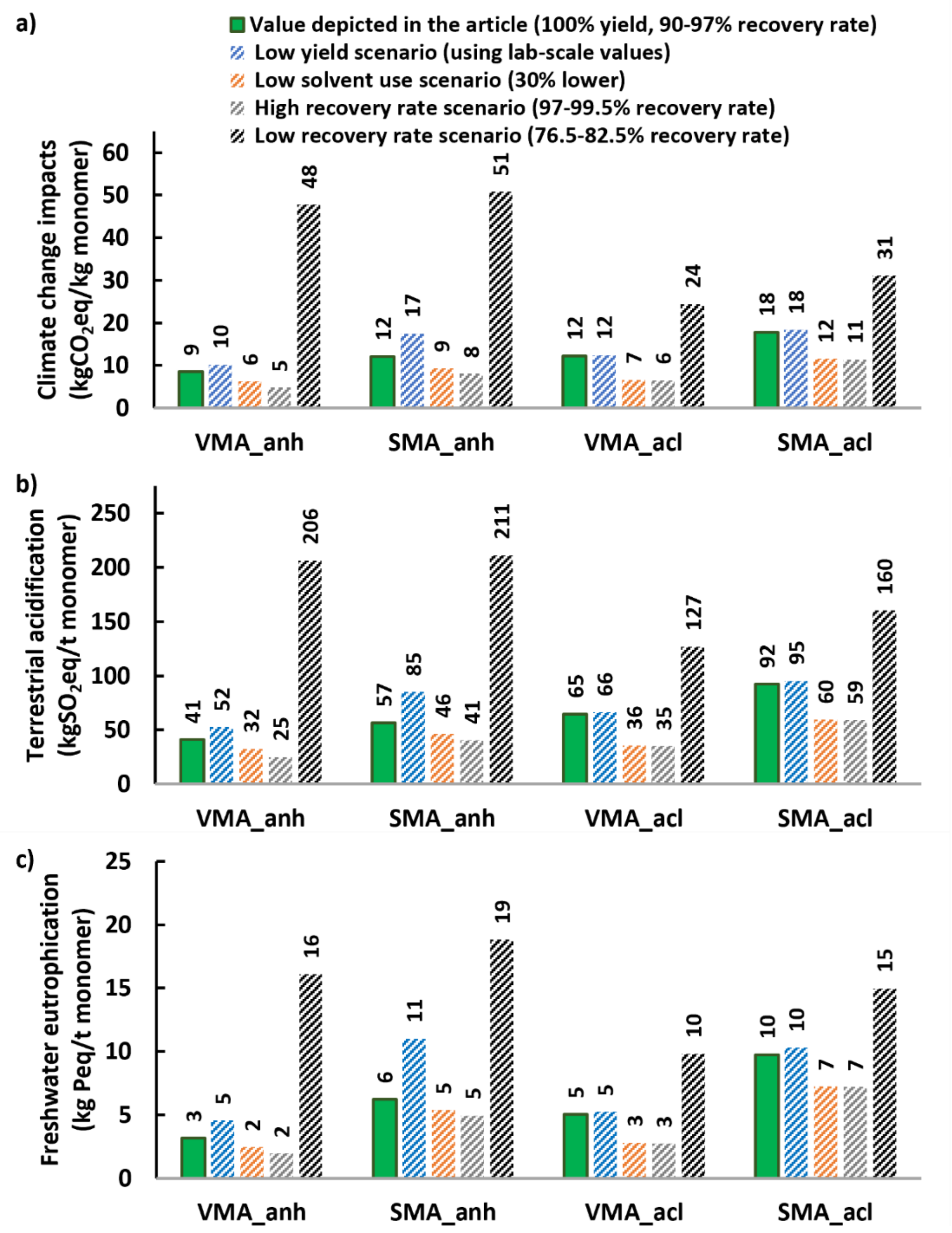

Figure S26. Sensitivity analysis of the environmental impacts of VMA-anh, VMA-acl, SMA-anh and SMA-acl. Climate change (a), terrestrial acidification (b), and freshwater eutrophication (c). Value depicted in the article refers to Figure 4 and Figure S25 (scaling-up).

Cell viability of monomers

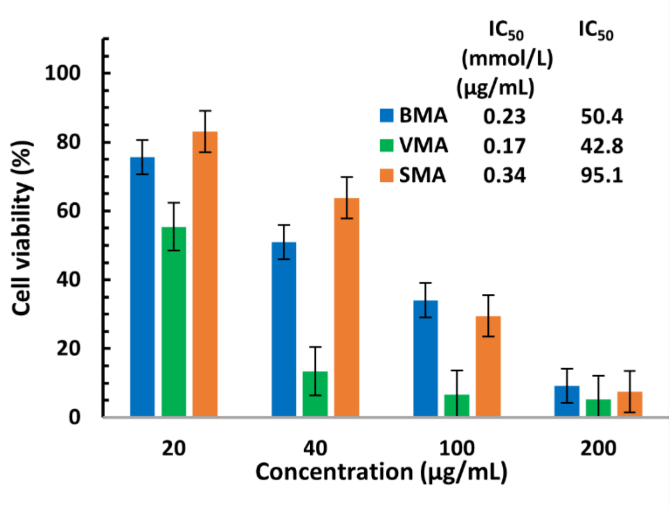

Figure S27. Cell viability and IC_{50} values of HeLa cells after exposure to the methacrylate monomers during 24 h.

References

[1] T. Mosmann, *J. Immunol. Methods* **1983**, *65*, 55–63, DOI:10.1016/0022-1759(83)90303-4.

[2] G. Ciapetti, E. Cenni, L. Pratelli, A. Pizzoferrato, *Biomaterials* **1993**, *14*, 359–364, DOI:10.1016/0142-9612(93)90055-7.

[3] K. G. Ozdemir, H. Yilmaz, S. Yilmaz, *J. Biomed. Mater. Res. - Part B Appl. Biomater.* **2009**, *90 B*, 82–86, DOI:10.1002/jbm.b.31256.

[4] N. R. D. de Souza, L. Matt, R. Sedrik, L. Vares, F. Cherubini, *Sustain. Prod. Consum.* **2023**, *43*, 319–332, DOI:10.1016/j.spc.2023.11.002.

[5] T. F. Stocker, D. Qin, G.-K. Plattner, M. Tignor, S. K. Allen, J. Boschung, A. Nauels, Y. Xia, V. (ed.). Bex, P. M. (ed.). Midgley, *IPCC, 2013: Climate Change 2013: The Physical Science Basis. Contribution of Working Group I to the Fifth Assessment Report of the Intergovernmental Panel on Climate Change*, Cambridge University Press, Cambridge, United Kingdom and New York, NY, USA, **2013**.

[6] M. A. J. Huijbregts, Z. J. N. Steinmann, P. M. F. Elshout, G. Stam, F. Verones, M. Vieira, M. Zijp, A. Hollander, R. van Zelm, *Int. J. Life Cycle Assess.* **2017**, *22*, 138–147, DOI:10.1007/s11367-016-1246-y.

[7] F. Piccinno, R. Hischier, S. Seeger, C. Som, *J. Clean. Prod.* **2018**, *174*, 283–295, DOI:10.1016/j.jclepro.2017.10.226.

[8] I. S. Modahl, E. Soldal, *The 2019 LCA of Products from Borregaard , Sarpsborg*, **2019**.

[9] N. Khwanjaisakun, S. Amornraksa, L. Simasatitkul, P. Charoensuppanimit, S.

Assabumrungrat, *Bioresour. Technol.* **2020**, *299*, DOI 10.1016/j.biortech.2019.122559, DOI:10.1016/j.biortech.2019.122559.

[10] E. Moreno Ruiz, D. FitzGerald, A. Symeonidis, D. Ioannidou, J. Müller, L. Valsasina, C. Vadenbo, N. Minas, T. Sonderegger, D. Dellenbach, **2021**.

[11] R. Sacchi, T. Terlouw, K. Siala, A. Dirnaichner, C. Bauer, B. Cox, C. Mutel, V. Daioglou, G. Luderer, *Renew. Sustain. Energy Rev.* **2022**, *160*, 112311, DOI:10.1016/j.rser.2022.112311.

[12] O. Fricko, P. Havlik, J. Rogelj, Z. Klimont, M. Gusti, N. Johnson, P. Kolp, M. Strubegger, H. Valin, M. Amann, T. Ermolieva, N. Forsell, M. Herrero, C. Heyes, G. Kindermann, V. Krey, D. L. McCollum, M. Obersteiner, S. Pachauri, S. Rao, E. Schmid, W. Schoepp, K. Riahi, *Glob. Environ. Chang.* **2017**, *42*, 251–267, DOI:10.1016/j.gloenvcha.2016.06.004.

[13] F. Vidal, E. R. van der Marel, R. W. F. Kerr, C. McElroy, N. Schroeder, C. Mitchell, G. Rosetto, T. T. D. Chen, R. M. Bailey, C. Hepburn, C. Redgwell, C. K. Williams, *Nature* **2024**, *626*, 45–57, DOI:10.1038/s41586-023-06939-z.

[14] P. Stegmann, V. Daioglou, M. Londo, D. P. van Vuuren, M. Junginger, *Nature* **2022**, *612*, 272–276, DOI:10.1038/s41586-022-05422-5.

[15] J. Le Nôtre, S. C. M. Witte-van Dijk, J. van Haveren, E. L. Scott, J. P. M. Sanders, *ChemSusChem* **2014**, *7*, 2712–2720, DOI:10.1002/cssc.201402117.

[16] A. Bohre, U. Novak, M. Grilc, B. Likozar, *Mol. Catal.* **2019**, *476*, 110520, DOI:10.1016/j.mcat.2019.110520.

[17] "PIK-POSTDAM, n.d. REMIND (REgional Model of Investment and Development).," can be found under https://www.pik-potsdam.de/en/institute/departments/transformation-pathways/models/remind/remind

[18] H. H. Khoo, L. L. Wong, J. Tan, V. Isoni, P. Sharratt, *Resour. Conserv. Recycl.* **2015**, *95*, 174–182, DOI:10.1016/j.resconrec.2014.12.013.
